# Mechanisms and Pathophysiological Significance of Insulin Resistance in Offspring With Intrauterine Growth Restriction Mediated by Hepatic GR/miR‐1224 Programming

**DOI:** 10.1002/advs.202510277

**Published:** 2025-09-03

**Authors:** Yongguo Dai, Xiaoling Guo, Pengxia Yu, Dingmei Zhang, Hao Kou, Hui Wang

**Affiliations:** ^1^ Department of Pharmacology School of Basic Medical Sciences Wuhan University Wuhan 430071 China; ^2^ Department of Pharmacy National Clinical Research Center for Geriatric Disorders Xiangya Hospital of Central South University Changsha 410008 China; ^3^ Department of Pharmacy Zhongnan Hospital of Wuhan University Wuhan 430071 China; ^4^ Hubei Provincial Key Laboratory of Developmentally Originated Disease School of Basic Medical Sciences Wuhan University Wuhan 430071 China

**Keywords:** advanced glycation end products, glucocorticoids, insulin resistance, miR‐1224, prenatal caffeine exposure

## Abstract

Insulin resistance (IR) has fetal origin and gender preference. Here we traced glucose‐insulin phenotypical changes of intrauterine growth restriction (IUGR) female offspring at different ages and explore the mechanism. The IUGR model was established by prenatal caffeine exposure (PCE): dams were orally administered caffeine [(30, 120 mg(kg.d)^−1^] from gestational day 9 to 20. PCE offspring presented decreased basal serum glucose, insulin, and insulin resistance index, accompanied by improved glucose tolerance and insulin sensitivity. However, after postnatal week (PW) 36, all these changes were gradually reversed in age‐dependent manner and finally developed glucose intolerance and IR. Meanwhile, hepatic insulin signaling and glucose uptake were also gone from enhancement to inhibition. Mechanistically, a negative regulation of blood glucocorticoid on hepatic insulin signaling and glucose uptake via glucocorticoid receptor (GR)/miR‐1224 signaling in the PCE offspring was programmed in utero. Postnatally, the persistent decreased blood glucocorticoid and consequent elevated hepatic glucose uptake caused over‐accumulation of advanced glycation end products (AGEs). AGEs promoted inflammation by interacting with its receptor and conversely repressed hepatic insulin signaling and glucose uptake after PW36. Conclusively, PCE‐induced IUGR offspring exhibited a shift from insulin sensitization to IR, which may be related to hepatic insulin signaling changes via GR/miR‐1224 programming.

## Graphical abstract

This work found that early‐stage insulin sensitization but late‐stage insulin resistance in prenatal caffeine exposure (PCE)‐ induced intrauterine growth restriction (IUGR) offspring may be related to the hepatic AGEs accumulation via intrauterine GR/miR‐1224 programming by glucocorticoids.

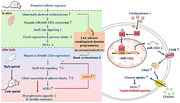

## 1 Introduction

Insulin resistance (IR) is defined physiologically as a state of decreased reactivity or sensitivity of insulin target tissues to high insulin levels and is considered a pathogenic driving factor for various metabolic disorders, including type two diabetes, non‐alcoholic fatty liver disease (NAFLD), and metabolic syndrome.^[^ ^1^, ^2^ ^]^ Over the past few decades, the incidence of IR and related diabetes has been steadily increasing. Traditional views attribute this phenomenon to genetic susceptibility and lifestyle changes as the main pathophysiological factors. However, increasing evidence suggests that some “adverse” factors during early developmental periods of individuals also play a role.^[^ ^3^ ^]^ Epidemiological and laboratory studies have confirmed that offspring with intrauterine growth restriction (IUGR) or low birth weight (LBW) caused by various adverse prenatal environments, such as maternal diseases, unhealthy lifestyles, and exposure to xenobiotics, are more prone to develop IR and metabolic disorders like type two diabetes.^[^ ^4^, ^5^, ^6^, ^7^ ^]^ Interestingly, unlike the direct observation of IR in different IUGR model offspring at different time points,^[^ ^8^, ^9^, ^10^, ^11^ ^]^ a longitudinal cohort study of LBW children showed that insulin sensitivity increased at the age of two and transitioned to IR at the age of four.^[^ ^12^ ^]^ Unfortunately, a comprehensive understanding of the systemic changes in glucose metabolism and function throughout the entire lifespan after birth and the underlying mechanisms in offspring with IUGR caused by adverse prenatal environments has not been fully elucidated.

One of the important causes of IR is the dysfunction of the insulin receptor (InsR) and downstream signaling in insulin target tissues.^[^ ^1^, ^13^, ^14^ ^]^ InsR is a receptor tyrosine kinase that, upon binding with insulin, leads to phosphorylation of insulin receptor substrates, thereby activating phosphoinositide 3‐kinase (PI3K) and protein kinase B (PKB, namely Akt), and subsequently activating downstream pathways.^[^ ^15^, ^16^ ^]^ This entire signaling cascade is crucial for glucose mobilization and transport. As a vital target tissue for insulin, the liver plays a pivotal role in glucose metabolism. After secretion from the pancreas, insulin reaches the liver as the first organ, where it regulates glucose storage and processing according to the body's requirements. Inhibition of the hepatic insulin signaling pathway (or hepatic IR) is one of the main causes of systemic IR.^[^ ^17^ ^]^ Several mechanisms regulating hepatic insulin signaling have been reported. Increasing evidence suggests that advanced glycation end products (AGEs) can impair insulin sensitivity and affect glucose homeostasis by binding to their receptor (RAGE).^[^ ^18^, ^19^ ^]^ AGEs are a group of stable and irreversible end products generated through certain reactions between reducing sugars and macromolecules such as proteins, lipids, or nucleic acids. Once formed, they are difficult to degrade and accumulate in the body with age, gradually altering the morphology and function of tissues and organs, and inducing related diseases.^[^ ^20^, ^21^, ^22^ ^]^ It has been reported that birth weight (adjusted for gestational age) is negatively correlated with levels of skin autofluorescence AGEs.^[^ ^23^ ^]^ Therefore, we speculate that the occurrence of IR in offspring with IUGR or LBW may be associated with AGEs accumulation.

Chronic stress refers to nonspecific systemic responses that occur in the body due to prolonged exposure to various internal and external stimuli. The harmful effects of chronic stress experienced by women during pregnancy on their offspring are increasingly evident. Caffeine, the most widely consumed psychoactive substance worldwide, is known for its stimulating and fatigue‐reducing effects and is commonly found in coffee, tea, chocolate, and compound preparation.^[^ ^24^, ^25^, ^26^, ^27^ ^]^ Studies have shown that long‐term caffeine exposure can lead to elevated levels of blood glucocorticoid (GC), indicating a pronounced chronic stress state.^[^ ^28^ ^]^ With the increasing consumption of caffeine‐containing foods and drugs, caffeine has become one of the common sources of chronic stress in modern life. Although caffeine intake by adults has been shown to be beneficial for various conditions such as neurological disorders, cardiovascular diseases, liver diseases, and type 2 diabetes,^[^ ^29^, ^30^ ^]^ compelling evidence from epidemiological and animal studies reveals multiple adverse effects caused by maternal caffeine exposure during pregnancy, even at previously considered “safe” doses.^[^ ^31^, ^32^, ^33^ ^]^ Numerous large‐scale epidemiological studies have demonstrated a close relationship between caffeine intake during pregnancy and IUGR or LBW.^[^ ^34^, ^35^, ^36^ ^]^ Our previous research has also confirmed that prenatal caffeine exposure (PCE) can induce IUGR in rodent models.^[^ ^37^, ^38^, ^39^, ^40^, ^41^ ^]^ Furthermore, our own and other previous studies have shown that PCE disrupts glucose‐insulin homeostasis in adult offspring.^[^ ^42^, ^43^, ^44^ ^]^ However, the underlying mechanisms remain incompletely understood.

Epidemiological investigations have found a higher prevalence of insulin sensitivity abnormalities in females with LBW compared to males.^[^ ^45^ ^]^ Our previous studies have revealed significant progressive changes in insulin sensitivity and more pronounced phenotypes of chronic diseases (such as non‐alcoholic fatty liver disease and osteoarthritis) in female IUGR offspring induced by PCE.^[^ ^46^, ^47^ ^]^ In this study, based on a previously established stable PCE‐induced IUGR rat model,^[^ ^38^, ^40^, ^41^, ^48^ ^]^ we aim to systematically observe the characteristics of glucose metabolism and insulin sensitivity phenotypic changes at different postnatal stages in female offspring. By combining cellular models, we will further investigate the intrauterine programming mechanisms underlying changes in insulin sensitivity in female offspring after birth due to PCE and explore the hepatic mechanisms of IR from the perspective of AGEs accumulation. This study will contribute to a better understanding of the developmental toxicity and long‐term hazards of caffeine and provide theoretical and experimental evidence for elucidating the mechanisms underlying fetal‐originated IR and exploring early prevention and treatment strategies for the IR‐related metabolic disorders.

## 2 Results

### 2.1 PCE Female Offspring Exhibited Dynamic Changes in Glucose Metabolic Homeostasis and Hepatic Insulin Sensitivity with Age

**Figure 1:**
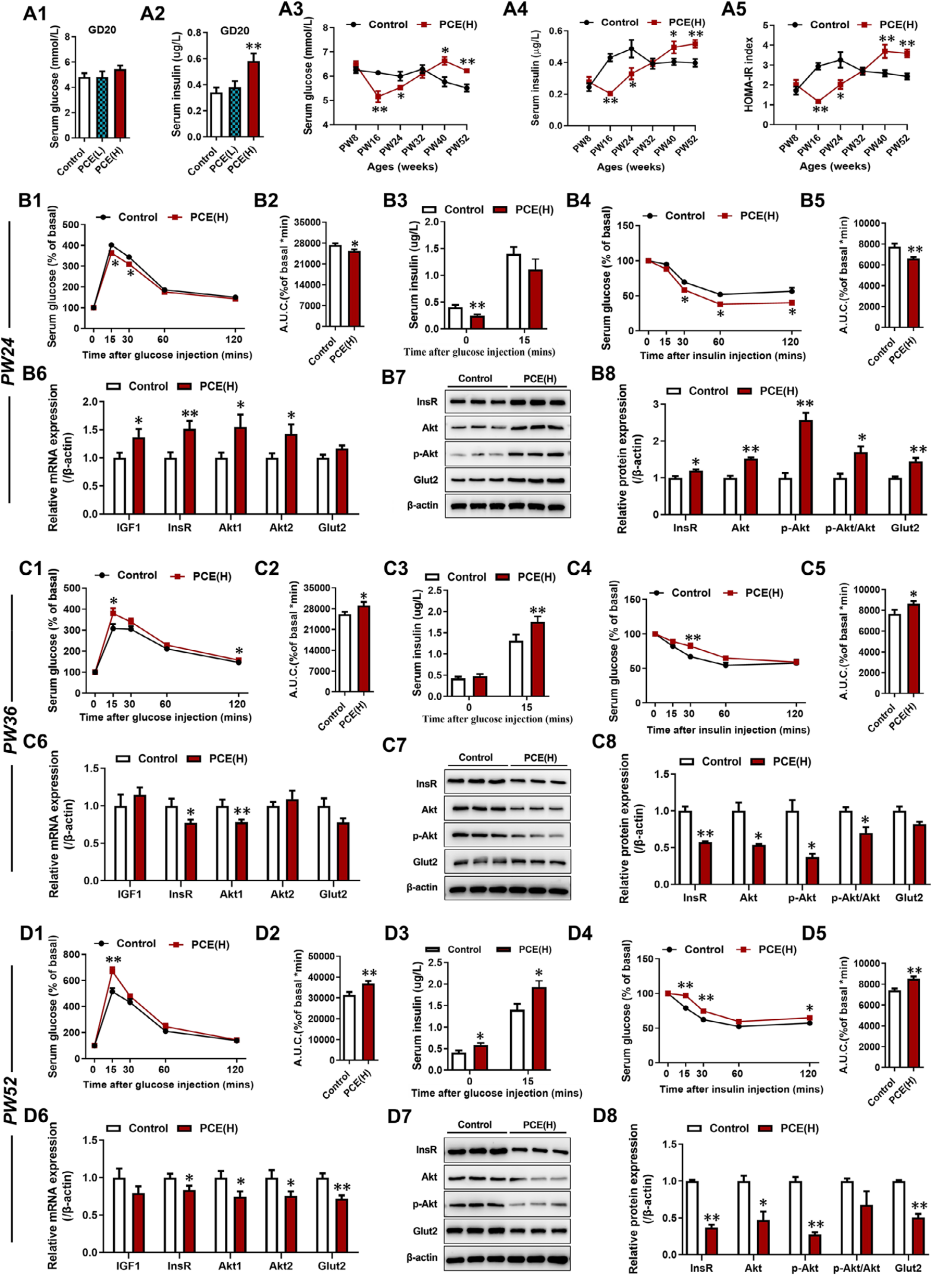
Dynamic changes of glucose metabolic homeostasis and hepatic insulin sensitivity in female offspring rats induced by PCE. (A1) Fasting serum glucose levels on GD20; (A2) Fasting serum insulin levels on GD20; (A3) Fasting serum glucose levels from PW8 to PW52; (A4) Fasting serum insulin levels from PW8 to PW52; (A5) HOMA‐IR index from PW8 to PW52; (B1, B2, C1, C2, D1, D2) Normalized blood glucose levels during IPGTT and corresponding AUC in PW24, PW36 and PW52; (B3, C3, D3) Blood insulin levels at 0 and 15 min during IPGTT in PW24, PW36 and PW52; (B4, B5, C4, C5, D4, D5) Normalized blood glucose levels during IPITT and corresponding AUC in PW24, PW36 and PW52; (B6, B7, C6, C7, D6, D7) Protein expression of hepatic InsR, Akt, p‐Akt‐S473 and Glut2 in PW24, PW36, and PW52. The data are shown as mean ± S.E.M., n = 3 for protein expression assay, n = 12 for other detection. Two‐tailed unpaired Student's t‐test (B2, B5, B7, C2, C5, C7, D2, D5, D7), One‐way ANOVA with Dunnett's post‐hoc test (A1, A2), Two‐way ANOVA for repeated measures followed by Bonferroni's post‐hoc test (A3‐5, B1, B3, B4, C1, C3, C4, D1, D3, D4). ^*^ *P<*0.05, ^**^ *P<*0.01 versus control. GD, gestational day; PW, postnatal week; HOMA‐IR, homeostasis model assessment of insulin resistance; IPGTT, intraperitoneal glucose tolerance test; IPITT, intraperitoneal insulin tolerance test; AUC, area under the curve; InsR, insulin receptor; Akt, AKT serine/threonine kinase; p‐Akt, phosphorylated Akt; Glut2, glucose transporter type 2; PCE(L), prenatal caffeine exposure [30 mg(kg·d)^−1^]; PCE(H), prenatal caffeine exposure [120 mg(kg·d)^−1^].

Firstly, we assessed the baseline of glucose‐insulin homeostasis in female offspring rats of PCE at different time points before (GD20) and after birth (PW8‐52) to evaluate the state and function of glucose metabolism. Compared to the control group, the baseline serum glucose levels in fetal rats of the PCE group showed no significant changes at different doses on GD20 (**Figure** **1**A1), while basal serum insulin levels were increased in the high‐dose group (Figure 1A2). At PW8, there were no significant changes in fasting serum glucose and insulin levels, as well as HOMA‐IR index, in PCE offspring rats (Figure 1A3–A5), but they were decreased at PW16 and PW24 (Figure 1A3–A5), recovered to normal levels at PW32, and significantly increased at PW40 and PW52 (Figure 1A3–A5). After glucose loading, compared to the control group, glucose tolerance in PCE offspring rats was enhanced at PW24 (Figure 1B1, B2), and there was no significant change in serum insulin levels at 15 min after glucose stimulation (Figure 1B3). However, glucose tolerance in PCE offspring rats was significantly decreased at PW36 and PW52 (Figure 1C1, C2, 1D1, D2), and serum insulin levels at 15 min after glucose stimulation were significantly increased (Figure 1C3, D3). These data indicate that despite the gradual recovery and compensatory increase in insulin production during the early stage, glucose metabolic function in PCE offspring rats gradually deteriorated with age and fell to maintain glucose homeostasis, eventually leading to hyperglycemia.

Next, we further evaluated the utilization and sensitivity of insulin in PCE offspring rats at both phenotypic and gene regulatory levels. In response to exogenous insulin load, compared to the control group, insulin sensitivity in PCE offspring rats was enhanced at PW24 (Figure 1B4, B5), while it was significantly decreased at PW36 and PW52 (Figure 1C4, C5, D4, D5). Liver plays a crucial role in the regulation of blood glucose levels. Compared to the control group, mRNA and/or protein expression levels of key markers in the hepatic insulin signaling pathway (including InsR, p‐Akt‐S473, Akt) and the major glucose uptake transporter Glut2 were increased at PW24 in PCE offspring rats (Figure 1B6, B7; Figure S2B, Supporting Information). At PW36, the hepatic insulin signaling pathway was suppressed in PCE offspring rats (Figure 1C6, C7; Figure S2C, Supporting Information), and there were no significant changes in Glut2 expression (Figure 1C6, C7; Figure S2C, Supporting Information). Furthermore, at PW52, their expression levels were decreased in the livers of PCE offspring rats (Figure 1D6,D7, Figure S2D, Supporting Information). The mRNA expression of key enzymes involved in hepatic gluconeogenesis (*Pck1* and *G6pc*) was significantly increased at PW24, PW36, and PW56 (Figure S3, Supporting Information). Additionally, compared to the control group, the mRNA expression of hepatic insulin‐like growth factor 1 (*Igf1*) in the liver of PCE offspring rats was elevated at PW24 (Figure S2B, Supporting Information), and restored to normal levels at PW36 and PW52 (Figure S2C,D, Supporting Information). These results indicated that hepatic insulin sensitivity in PCE offspring rats gradually decreased with age, consistent with the changes in systemic insulin sensitivity.

### 2.2 High‐Endogenous GC Levels via GR/miR‐1224 Inhibited Hepatic Insulin Signaling and Glucose Uptake in PCE Female Fetal Rats

**Figure 2:**
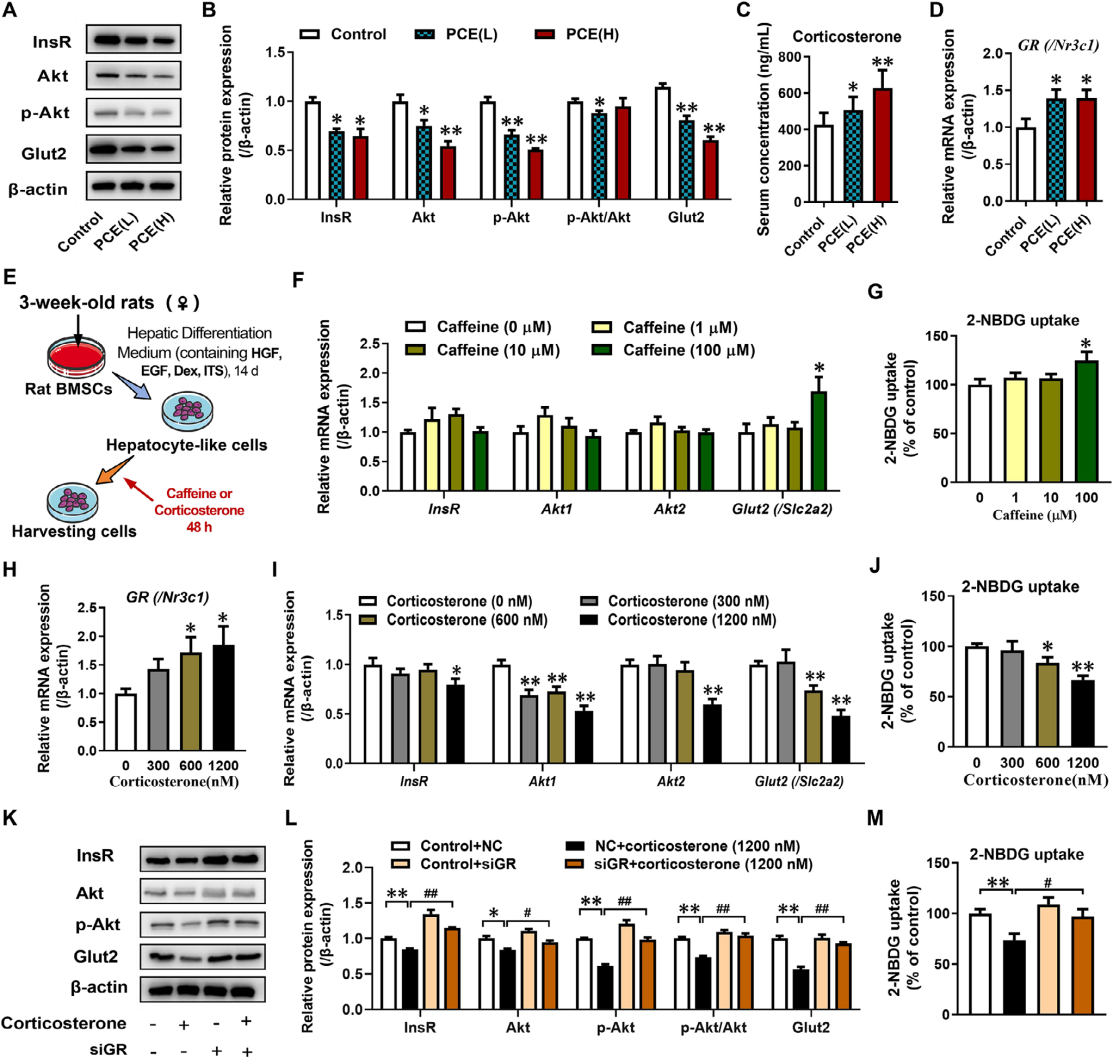
Impaired hepatic insulin signaling pathway and glucose transport function in PCE female fetal rats were attributed to excessive endogenous glucocorticoid levels in utero, rather than a direct action of caffeine. A,B) Protein expression of hepatic InsR, Akt, p‐Akt‐S473 and Glut2 on GD20; C) Serum corticosterone levels on GD20; D) Hepatic *GR* (/*Nr3c1*) mRNA expression on GD20; E) Schematic diagram of caffeine or high concentration of corticosterone treatment in hepatocyte‐like cells differentiated from rat BMSCs; F,I) mRNA expression of *InsR, Akt1, Akt2* and *Glut2* (/*Slc2a2*) in hepatocyte‐like cells differentiated from rat BMSCs after treatment with caffeine or high concentration of corticosterone; G,J) 2‐NBDG uptake in hepatocyte‐like cells differentiated from rat BMSCs after treatment with caffeine or high concentration of corticosterone; H) *GR* (/*Nr3c1*) mRNA expression in hepatocyte‐like cells differentiated from rat BMSCs after treatment with high concentration of corticosterone; K,L) Protein expression of hepatic InsR, Akt, p‐Akt‐S473 and Glut2 in hepatocyte‐like cells differentiated from rat BMSCs after treatment with siGR and/or high concentration of corticosterone; (M) 2‐NBDG uptake in hepatocyte‐like cells differentiated from rat BMSCs after treatment with siGR and/or high concentration of corticosterone. The data are shown as mean ± S.E.M., n = 3 for protein expression assay, n = 6 for gene expression and 2‐NBDG uptake assay in vitro, n = 12 for other detection. One‐way ANOVA with Dunnett's post‐hoc test (B‐D, F‐J), One‐way ANOVA with Tukey's post‐hoc test (L, M). ^*^ *P*<0.05, ^**^ *P*<0.01 versus control; *^#^P*<0.05, ^##^ *P*<0.01 versus corticosterone‐treated group. BMSCs, bone marrow mesenchymal stem cells; GD, gestational day; InsR, insulin receptor; Akt, AKT serine/threonine kinase; p‐Akt, phosphorylated Akt; Glut2, glucose transporter type 2; *GR* (/*Nr3c1*), glucocorticoid receptor; 2‐NBDG, 2‐Deoxy‐2‐ [(7‐nitro‐2,1,3‐benzoxadiazol‐4‐yl) amino]‐D‐glucose; PCE(L), prenatal caffeine exposure [30 mg(kg·d)^−1^]; PCE(H), prenatal caffeine exposure [120 mg(kg·d)^−1^].

To investigate the fetal origin of altered hepatic insulin sensitivity in PCE offspring, we further assessed the impact of PCE on the hepatic insulin signaling pathway and glucose metabolism in fetal rats. Compared to the control group, the mRNA expression of *Igf1*, as well as the mRNA and protein expression of key markers (InsR, Akt, and p‐Akt‐S473) in the insulin signaling pathway, were significantly decreased in PCE fetal rat livers (**Figure** **2**A,B, Figure S2A, Supporting Information). Additionally, the mRNA and protein expression of hepatic Glut2 were significantly suppressed in PCE fetal rats (Figure 2A,B, Figure S2A, Supporting Information). Furthermore, PCE significantly promoted the mRNA expression of key enzymes involved in gluconeogenesis (*Pck1* and *G6pc*) (Figure S3, Supporting Information). These data indicated that PCE inhibited the hepatic IGF1/insulin signaling pathway in the fetal rats and may affect glucose metabolism.

In our previous study, we demonstrated that ≈61% of caffeine crosses the placenta and enters the fetal circulation, resulting in fetal caffeine concentrations reaching 155 ± 28 µµ (29±5 µgmL^−1^).^[^ ^46^, ^58^ ^]^ To confirm the direct effects of caffeine on the hepatic insulin signaling pathway and glucose uptake transport function in fetal rats, we established a model of the differentiation of rat BMSCs into hepatocyte‐like cells according to a previously reported method^[^ ^52^ ^]^ and treated the cells with different concentrations of caffeine (0, 1, 10, and 100 mµ) for 48 h (Figure 2E). The results showed that the mRNA expression of InsR, Akt1, and Akt2 in the cells was not significantly affected by caffeine treatment (Figure 2F). However, the *Glut2* mRNA expression and glucose uptake function were significantly increased after treatment with 100 mµ caffeine (Figure 2F,G). These findings were inconsistent with the in vivo results in fetal rats, suggesting that the inhibition of the hepatic insulin pathway and decreased glucose uptake in the liver of PCE fetal rats may not be directly related to caffeine and may comprise other influencing factors.

Caffeine is a common chronic stressor in modern life and can lead to elevated GC (i.e., corticosterone in rats, cortisol in humans) levels. Our previous study indicated that PCE fetal rats experience excessive exposure to maternal GC.^[^ ^59^ ^]^ Similarly, in this study, we observed significantly elevated levels of endogenous GC (i.e., corticosterone) in the serum of PCE fetal rats compared to the control group (Figure 2C). Additionally, the expression of hepatic *GR* (/*Nr3c1*) was significantly increased in PCE fetal rats (Figure 2D). Further in vitro studies using hepatocyte‐like cells differentiated from rat BMSCs showed that after 48 h of treatment with different concentrations of corticosterone (Figure 2E), without affecting cell viability (Figure S4, Supporting Information), corticosterone significantly increased *GR* (/*Nr3c1*) expression in a concentration‐dependent manner (Figure 2H) and decreased the mRNA expression of *InsR*, *Akt1*, *Akt2*, *Glut2*, as well as 2‐NBDG uptake (Figure 2I,J). These effects induced by high concentrations of corticosterone were significantly reversed after GR siRNA treatment (Figure 2K–M; Figure S5, Supporting Information). These findings suggested that high levels of endogenous GC during prenatal development activated GR, which contributes to the reduced hepatic insulin sensitivity and impaired glucose uptake in the liver of PCE fetal rats.

**Figure 3:**
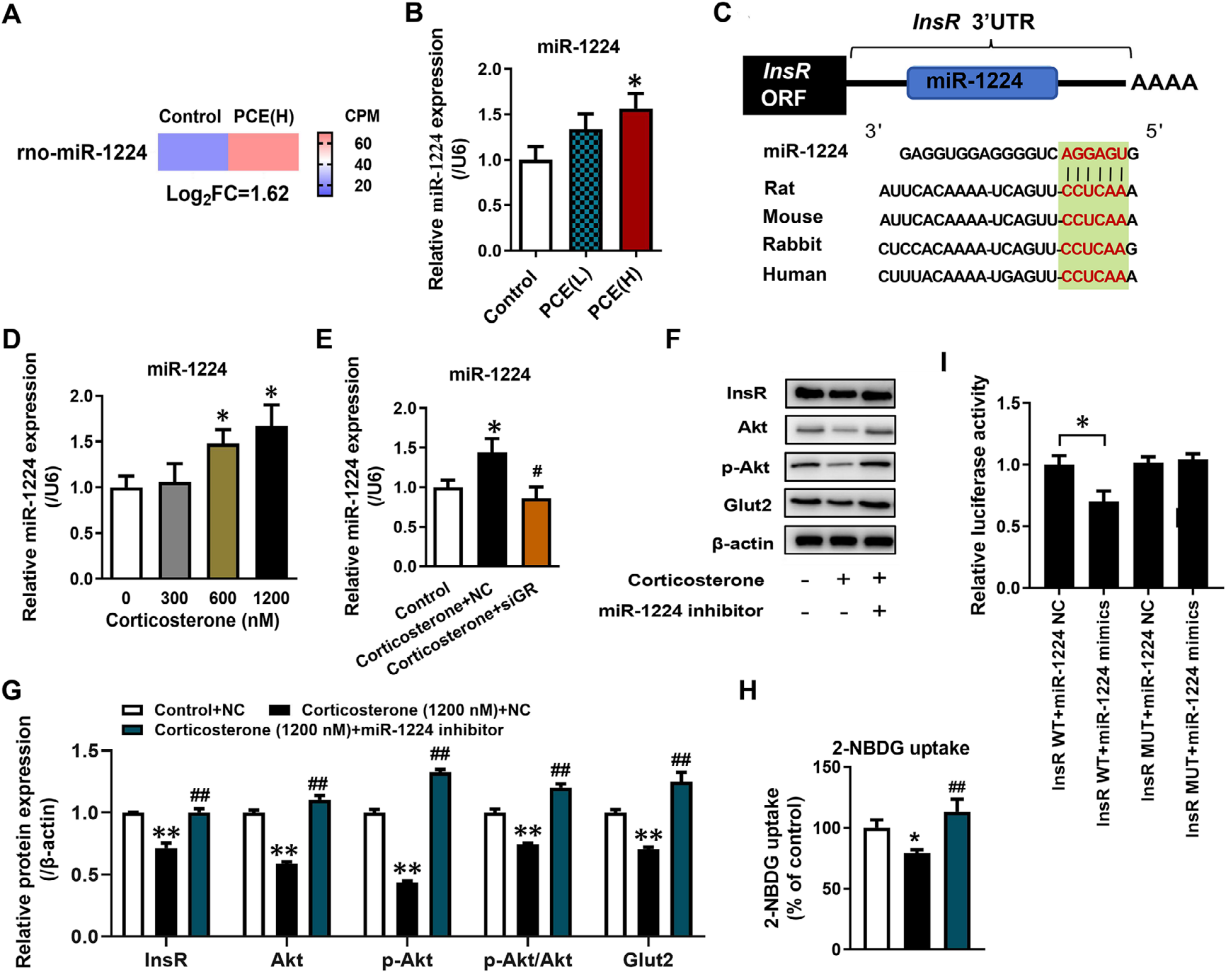
Hepatic GR/miR‐1224 mediated the regulation of hepatic insulin signaling pathway and glucose uptake function in PCE female fetal rats by excessive endogenous glucocorticoid. A, B) Hepatic miR‐1224 expression on GD20 by miRNA sequencing and RT‐qPCR; C) Graphic representation of the conserved miR‐1224 binding motifs within the 3′UTR of *InsR*; D, E) miR‐1224 expression in hepatocyte‐like cells differentiated from rat BMSCs after treatment with siGR and/or high concentration of corticosterone; F, G) Protein expression of InsR, Akt, p‐Akt‐S473 and Glut2 in hepatocyte‐like cells differentiated from rat BMSCs after treatment with miR‐1224 inhibitor and/or high concentration of corticosterone; H) 2‐NBDG uptake in hepatocyte‐like cells differentiated from rat BMSCs after treatment with miR‐1224 inhibitor and/or high concentration of corticosterone; I) Luciferase activity of the reporter constructs containing either WT or mutated 3′UTR of rat InsR after treatment with miR‐1224 mimics. The data are shown as mean ± S.E.M., n = 3 for protein expression assay, n = 6 for other detection. One‐way ANOVA with Dunnett's post‐hoc test (B, D), One‐way ANOVA with Tukey's post‐hoc test (E, G‐I). ^*^ *P<*0.05, ^**^ *P<*0.01 versus control; ^##^ *P<*0.01 versus corticosterone‐treated group. InsR, insulin receptor; Akt, AKT serine/threonine kinase; p‐Akt, phosphorylated Akt; BMSCs, bone marrow mesenchymal stem cells; Glut2, glucose transporter type 2; 2‐NBDG, 2‐Deoxy‐2‐ [(7‐nitro‐2,1,3‐benzoxadiazol‐4‐yl) amino]‐D‐glucose; PCE(L), prenatal caffeine exposure [30 mg(kg·d)^−1^]; PCE(H), prenatal caffeine exposure [120 mg(kg·d)^−1^].

As an epigenetic mechanism, miRNA is known to be involved in the programmed regulation of diseases under the influence of intrauterine factors.^[^ ^60^, ^61^ ^]^ We further assessed whether the inhibitory effect of intrauterine high‐endogenous glucocorticoids on the insulin signaling pathway and glucose uptake function in PCE fetal liver involves the participation of miRNAs. The expression of miRNAs in the liver of PCE fetal rats was evaluated through miRNA sequencing (**Figure** **3**A; Table S3, Supporting Information) and validated by RT‐qPCR (Figure 3B). Predictive analysis on the JASPAR website (https://jaspar.genereg.net/) revealed the presence of GR binding sites (Figure S6, Supporting Information) in the promoter region of the highly expressed miR‐1224 in the liver of PCE fetal rats (Figure 3B). Furthermore, the analysis on the TargetScan website (https://www.targetscan.org/vert_80/) indicated its potential targeting of *InsR* (Figure 3C). Consistent with in vivo studies, in hepatocyte‐like cells differentiated from rat BMSCs, increasing concentrations of corticosterone led to a concentration‐dependent upregulation of miR‐1224 expression (Figure 3D). Following treatment with GR siRNA, the upregulation of miR‐1224 expression induced by high concentrations of corticosterone was reversed (Figure 3E). Moreover, the inhibition of miR‐1224 by using miR‐1224 inhibitors significantly reversed the inhibitory effect of high concentrations of corticosterone on the insulin signaling pathway (InsR, p‐Akt/Akt, and Glut2 mRNA and protein expression) and glucose uptake function (Figure 3F–H; Figure S7A, Supporting Information). Additionally, miR‐1224 mimics and dual luciferase reporter gene assays further confirmed the direct regulatory effect of miR‐1224 on InsR expression (Figure 3I; Figure S7B,C, Supporting Information), which affects subsequent insulin signaling and glucose uptake function (Figure S7B,D, Supporting Information). These data suggested that the high expression of hepatic GR/miR‐1224 mediates the inhibitory effect of intrauterine high GC on the insulin signaling pathway and glucose uptake function in PCE fetal rat liver.

### 2.3 Low‐Endogenous GC Levels via GR/miR‐1224 Programed Enhancement of Hepatic Insulin Signaling and Glucose Uptake in Early Postnatal Stages of PCE Female Offspring

**Figure 4:**
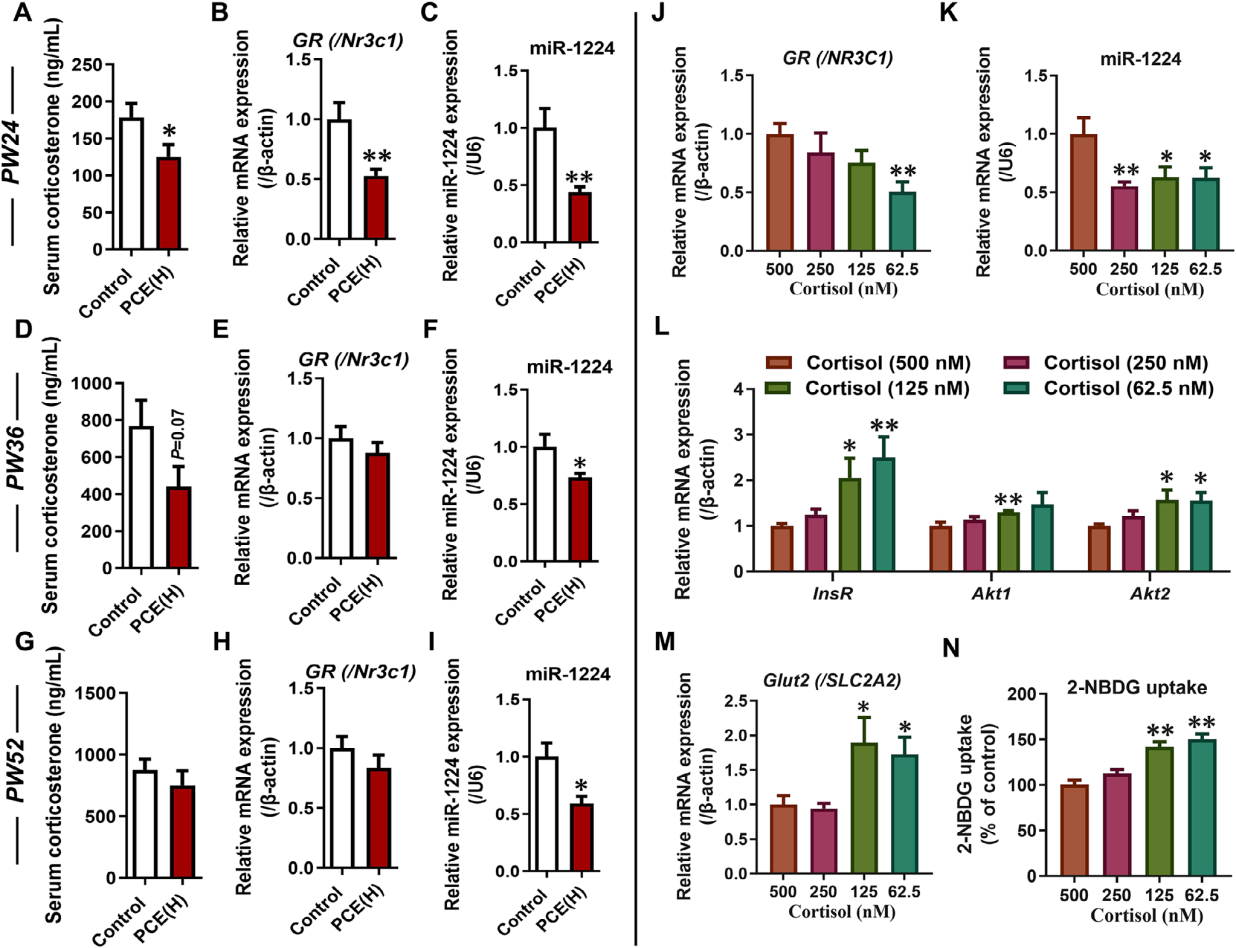
Low‐endogenous glucocorticoid levels were involved in programming the hepatic insulin signaling pathway and glucose uptake function of PCE female offspring rats at early postnatal stages via GR/miR‐1224. A, D, G) Serum corticosterone levels in PW24, PW36, and PW52; (B, E, H) Hepatic *GR* (/*Nr3c1*) mRNA expression in PW24, PW36 and PW52; C, F, I) Hepatic miR‐1224 expression in PW24, PW36 and PW52; J, L, M) mRNA expressions of *GR* (/*Nr3c1*), *InsR*, *Akt1*, *Akt2* and *Glut2* (/*Slc2a2*) in HepG2 cells after treatment with lower than physiological levels of cortisol; K) miR‐1224 expression in HepG2 cells after treatment with lower than physiological levels of cortisol; N) 2‐NBDG uptake in HepG2 cells after treatment with lower than physiological levels of cortisol. The data are shown as mean ± S.E.M., n = 12 for animal assay, n = 6 for other detection. Two‐tailed unpaired Student's t‐test (A, D‐I), Two‐tailed unpaired Student's t‐test with Welch's correction (B, C), One‐way ANOVA with Dunnett's post‐hoc test [J, K, L (Akt1 and Akt2), N], One‐way Brown‐Forsythe ANOVA with Dunnett's T3 post‐hoc test [L (InsR), M). *P*<*0.05, ^**^ *P<*0.01 versus control (/500 nM cortisol). *GR* (/*Nr3c1*), glucocorticoid receptor; *InsR*, insulin receptor; *Akt1*, AKT serine/threonine kinase 1; *Akt2*, AKT serine/threonine kinase 2; *Glut2* (/*Slc2a2*), glucose transporter type 2; 2‐NBDG, 2‐Deoxy‐2‐ [(7‐nitro‐2,1,3‐benzoxadiazol‐4‐yl) amino]‐D‐glucose; PCE(H), prenatal caffeine exposure [120 mg(kg·d)^−1^].

Contrary to the changes observed in utero, PCE female offspring rats exhibited decreased levels of endogenous GC in the serum and reduced hepatic GR (/Nr3c1) expression at PW24 (**Figure** **4**A,B), accompanied by a decrease in miR‐1224 expression (Figure 4C). Concurrently, the insulin signaling pathway and Glut2 expression in the liver also showed corresponding opposite changes (Figure 1B6,B7; Figure S2B, Supporting Information). Similarly, in an in vitro experiment using the human hepatic cell line HepG2, treatment with cortisol (human endogenous GC) at levels lower than physiological concentrations resulted in decreased expression of *GR* (/*Nr3c1*) and miR‐1224 in HepG2 cells with decreasing corticosterone levels (Figure 4J,K), as well as enhanced insulin signaling pathway and glucose uptake function (Figure 4L–N). These findings further suggested that hepatic insulin sensitivity and glucose uptake capacity in PCE offspring rats can be influenced by changes in endogenous GC levels. It is worth noting that with increasing age at PW36 and PW52, endogenous corticosterone levels and hepatic *GR* (/*Nr3c1*) expression in PCE offspring gradually returned to normal levels (Figure 4D,I,G,H), while miR‐1224 expression continued to decrease (Figure 4F,I), and the insulin signaling pathway and glucose uptake function shifted to an inhibited state (Figure 1C6,7,D6, D7; Figure S2C,D, Supporting Information). This suggested the involvement of additional factors in inducing late‐life IR in PCE female offspring rats.

### 2.4 Accelerated Accumulation of AGEs in PCE Female Offspring Rats Promoted Inhibition of the Hepatic Insulin Signaling and Glucose Uptake

**Figure 5:**
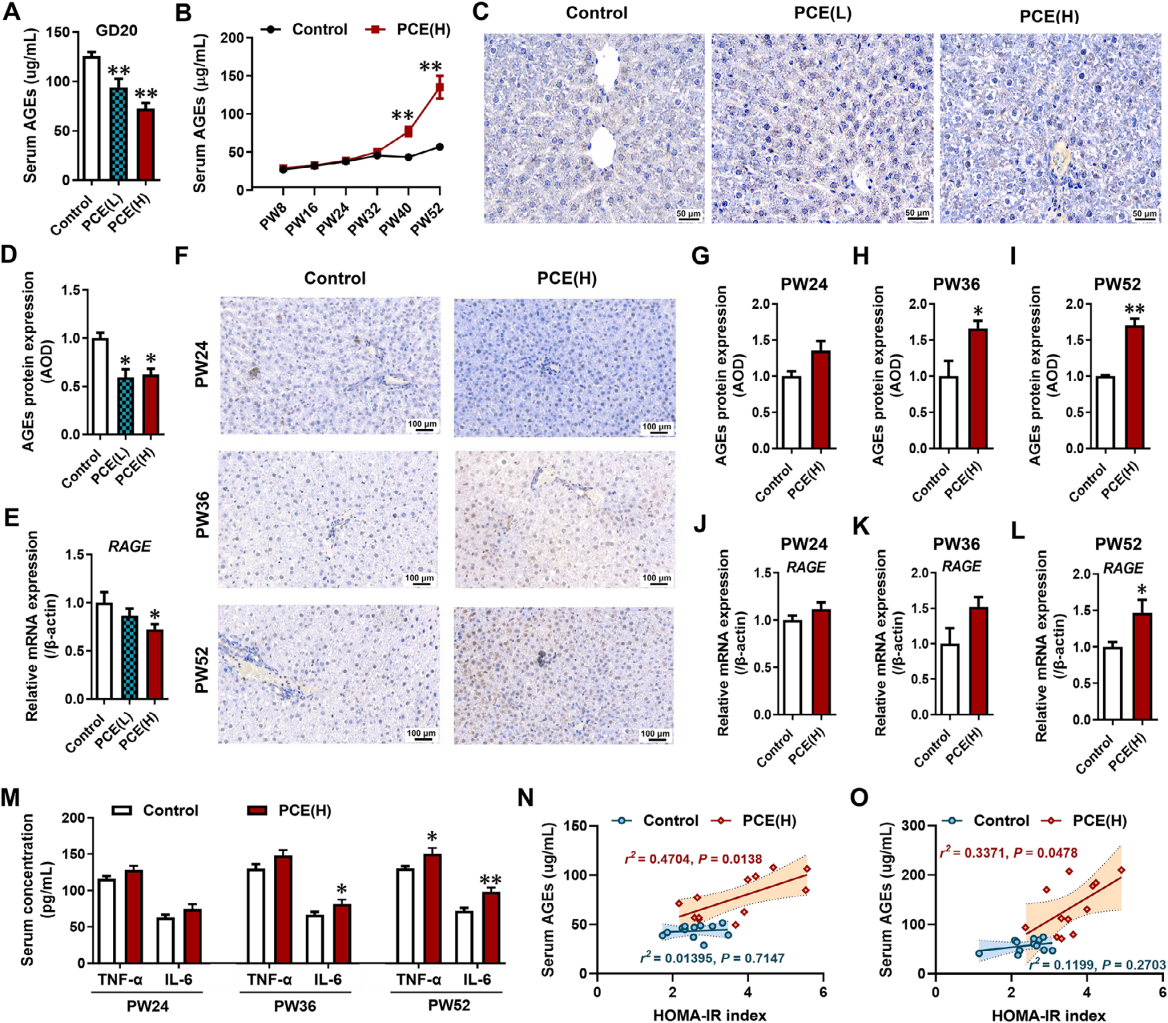
Age‐dependent accumulation of AGEs in female offspring rats induced by PCE. A) Serum AGEs levels in the GD20 fetuses; B) Serum AGEs levels in offspring from PW8 to PW52; C, D) Hepatic AGEs content on GD20, scale bar: 50 µm; E) Hepatic RAGE mRNA expression in the GD20 fetuses; F–I) Hepatic AGEs content in the PW24, PW36 and PW52 offspring, scale bar: 100 µm; J, K, L) Hepatic RAGE mRNA expression in the PW24, PW36, and PW52 offspring; M) Hepatic TNF‐α and IL‐6 mRNA expression in the PW24, PW36, and PW52 offspring; N, O) Correlation between HOMA‐IR index and serum AGEs levels in the PW40 and PW52 offspring. The data are shown as mean ± S.E.M., n = 3 for hepatic AGEs content, n = 12 for other detection. Two‐tailed unpaired Student's t‐test (G‐M), One‐way ANOVA with Dunnett's post‐hoc test (A, D, E), Two‐way ANOVA for repeated measures followed by Bonferroni's post‐hoc test (B). *P<0.05, **P<0.01 versus control. AGEs, advanced glycation end‐products; RAGE, AGEs receptor; GD, gestational day; PW, postnatal week; HOMA‐IR, homeostasis model assessment of insulin resistance; IL‐6, interleukin 6; TNF‐α, tumor necrosis factor α; PCE(L), prenatal caffeine exposure [30 mg(kg·d)−1]; PCE(H), prenatal caffeine exposure [120 mg(kg·d)−1].

Excessive accumulation of AGEs has been reported to be associated with impaired insulin signaling pathway and IR.^[^ ^62^, ^63^ ^]^ Interestingly, compared to the control group, PCE offspring rats exhibited a significant progressive increase in serum AGEs levels in the late stages (PW40 and PW52) (**Figure** **5**B), despite a decrease in levels during prenatal development (Figure 5A) and no significant changes during the early postnatal stages (PW8, PW16, PW24, PW32) (Figure 5B). Similarly, the hepatic AGEs content and its receptor *RAGE* mRNA expression in PCE offspring rats were decreased during intrauterine development (Figure 5D,E) but showed varying degrees of increase at PW36 and PW52 (Figure 5F–L). Furthermore, compared to the control group, PCE offspring exhibited elevated levels of serum TNF‐α and IL‐6 at PW36 and PW52 to different extents (Figure 5M). Additionally, pearson correlation analysis revealed a significant positive correlation between serum AGEs levels and HOMA‐IR index in PCE offspring rats at PW40 and PW52 (Figure 5N,O). These findings suggested that the progressive accumulation of AGEs may contribute to the development of late‐life IR in PCE offspring rats.

**Figure 6:**
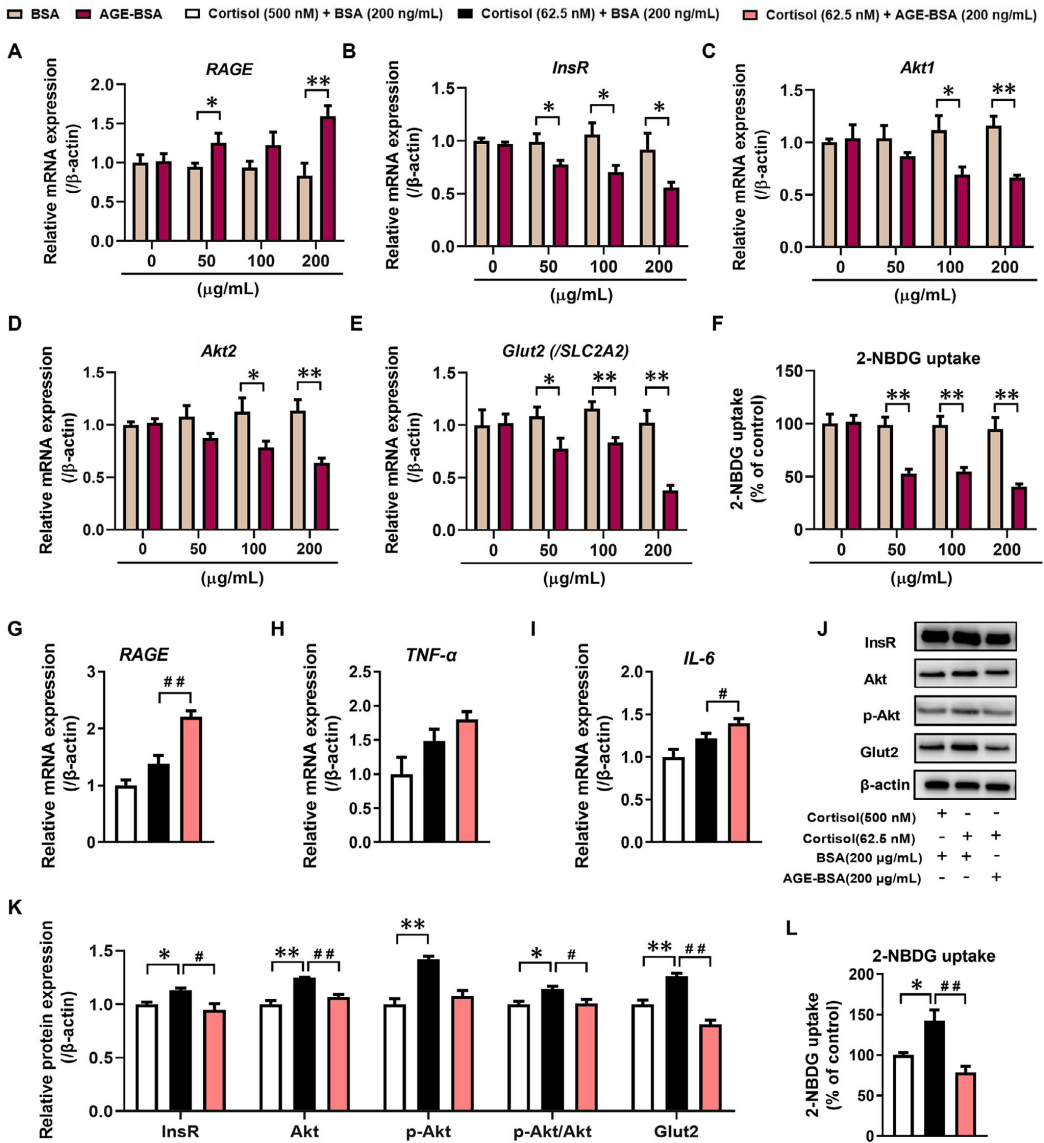
AGEs inhibited the enhancement of the insulin signaling pathway and glucose uptake in HepG2 cells caused by lower than physiological concentrations of cortisol. A–E) mRNA expressions of RAGE, InsR, Akt1, Akt2 and Glut2 (/Slc2a2) in HepG2 cells after treatment with AGE‐BSA; F) 2‐NBDG uptake in HepG2 cells after treatment with AGE‐BSA; G–I) mRNA expressions of RAGE, TNF‐α and IL‐6 in HepG2 cells after treatment with AGE‐BSA under lower than physiological concentrations of cortisol; J, K) Protein expression of hepatic InsR, Akt, p‐Akt‐S473 and Glut2 in HepG2 cells after treatment with AGE‐BSA under lower than physiological concentrations of cortisol; L) 2‐NBDG uptake in HepG2 cells after treatment with AGE‐BSA under lower than physiological concentrations of cortisol. The data are shown as mean ± S.E.M., n = 3 for protein expression assay, n = 6 for other detection. Two‐tailed unpaired Student's t‐test (A‐F), One‐way ANOVA with Tukey's post‐hoc test (G, H, I, K, L). *P<0.05, **P<0.01 versus control (BSA and 500 nM cortisol) group; #P<0.05, ##P<0.01 versus cortisol (62.5 nM)‐treated group. RAGE, AGEs receptor; InsR, insulin receptor; Akt1, AKT serine/threonine kinase 1; Akt2, AKT serine/threonine kinase 2; p‐Akt, phosphorylated Akt; Glut2, glucose transporter type 2; 2‐NBDG, 2‐Deoxy‐2‐ [(7‐nitro‐2,1,3‐benzoxadiazol‐4‐yl) amino]‐D‐glucose; IL‐6, interleukin 6; TNF‐α, tumor necrosis factor α.

To confirm the effects of AGEs on the insulin signaling pathway and inflammation‐related markers, we treated HepG2 cells with different concentrations of AGE‐BSA (50, 100, 200 µgmL^−1^) and used corresponding concentrations of BSA (50, 100, 200 µgmL^−1^) as controls. The results showed that compared to their respective control groups, treatment with AGE‐BSA led to an increase in *RAGE* mRNA expression in HepG2 cells (**Figure** **6**A), and a concentration‐dependent decrease in mRNA expression of insulin signaling pathway components (*InsR*, *Akt1*, *Akt2*) (Figure 6B,D). Furthermore, treatment with AGE‐BSA resulted in a concentration‐dependent decrease in *Glut2* expression and glucose uptake levels in HepG2 cells (Figure 6E,F). These results indicated that the AGEs‐RAGE signaling pathway can exert inhibitory effects on the insulin signaling pathway and glucose uptake function in HepG2 cells.

Given that postnatal PCE offspring exhibited enhanced insulin signaling pathway and glucose uptake function in the early stages due to decreased endogenous glucocorticoid levels, we further investigated the impact of AGE‐BSA (200 µgmL^−1^) on the outcomes induced by sub‐physiological concentrations of cortisol (62.5 nM) in HepG2 cells. Under conditions of sub‐physiological cortisol concentrations, treatment with AGE‐BSA significantly increased *RAGE* mRNA expression (Figure 6G), accompanied by an induction of the inflammatory cytokine *IL‐6* mRNA expression (Figure 6I). Additionally, AGE‐BSA treatment markedly inhibited the increase in insulin signaling pathway, Glut2 expression, and 2‐NBDG uptake induced by sub‐physiological cortisol levels (Figure 6J–L; Figure S8, Supporting Information). And the above effects caused by AGEs were significantly blocked by RAGE antagonist FPS‐ZM1 (Figure S9, Supporting Information). Taken together, these findings suggested that the accumulation of AGEs promotes the inhibition of the insulin signaling pathway and decreases glucose uptake function in the liver of PCE offspring rats.

## 3 Discussion

As a chronic stressor, caffeine intake during pregnancy has attracted increasing attention due to its safety concerns. It has been demonstrated as a significant adverse factor contributing to compromised outcomes such as IUGR/LBW and organ malformation.^[^ ^41^, ^59^, ^64^, ^65^, ^66^ ^]^ Building upon our previously established PCE‐induced IUGR rat model,^[^ ^38^, ^40^, ^41^ ^]^ this study focused on investigating the age‐related changes in glucose metabolism and insulin sensitivity, as well as elucidating the underlying programming mechanism. The main findings of this study included the following: ①Glucose tolerance and insulin sensitivity in PCE offspring rats gradually declined with increasing age after an initial enhancement; ②GR and miR1224 programming in the liver, dependent on GC levels, affected the insulin signaling pathway and glucose uptake function in PCE offspring during the prenatal and early postnatal stages; ③Accumulation of AGEs in PCE offspring contributed to the eventual development of IR in the late postnatal stage.

### 3.1 Age‐Related Decline in Glucose Tolerance and Insulin Sensitivity in PCE Offspring

In this study, maternal caffeine intake was identified as an adverse factor during pregnancy that contributes to the development of IR in the offspring. Specifically, the PCE group exhibited a gradual decline in glucose tolerance and insulin sensitivity in the late stage (after PW36), accompanied by hyperglycemia and elevated blood insulin levels, indicative of the onset of IR. Notably, similar to the phenotypes observed in our previous model with maternal caffeine exposure during GD11‐20,^[^ ^42^ ^]^ the present study also revealed an increase in glucose tolerance and insulin sensitivity in the early postnatal stage (before PW24) of PCE‐induced IUGR offspring rats. However, with advancing age, glucose tolerance and insulin sensitivity in PCE offspring gradually diminished, leading to the development of IR. This age‐related progression of changes has also been observed in IUGR/LBW offspring of different species by other researchers.^[^ ^12^, ^67^, ^68^ ^]^ For instance, insulin sensitivity increased in LBW children at the age of two but transitioned to IR at the age of four;^[^ ^12^ ^]^ and IUGR enhanced insulin sensitivity in IUGR piglets at 49 days of age but resulted in the opposite outcome at 105 days of age.^[^ ^67^ ^]^ These data suggested that the age‐related transition in glucose metabolic homeostasis and insulin sensitivity may be a common feature in IUGR offspring. However, many previous studies have only observed the outcomes of glucose metabolic capacity and insulin sensitivity at a single (or limited) time point(s), which may have led to the oversight of dynamic changes in glucose metabolic capacity and insulin sensitivity. For example, in a recent study by Gomes et al., higher insulin sensitivity was observed in offspring exposed to glyphosate during pregnancy and lactation, and an increase in glucose tolerance was observed on PD60, followed by normalization on PD143.^[^ ^69^ ^]^ The phenomenon observed in that study is like what we observed in the early postnatal stage of PCE offspring rats. Unfortunately, the study did not track the late‐stage changes in glucose metabolic capacity and insulin sensitivity in offspring exposed to glyphosate during maternal pregnancy and lactation, which might have led to the unrecognized occurrence of IR. Therefore, these findings also emphasized the need for comprehensive assessments of the impact of a specific factor during pregnancy on offspring glucose metabolism at multiple time points to avoid being misled by transient illusions and to prevent “looking at a leopard through a tube” that may hinder a thorough understanding.

### 3.2 Hepatic Insulin Signaling and Glucose Uptake Function Programmed by GC‐Dependent GR/miR‐1224 in PCE Offspring During Prenatal and Early Postnatal Stages

Epidemiological investigations in human populations and studies using animal models have demonstrated that adverse stimuli during fetal development have long‐term metabolic consequences in the offspring, suggesting the presence of intrauterine programming effects.^[^ ^70^, ^71^, ^72^ ^]^ Intrauterine programming refers to the process by which insults during intrauterine development lead to permanent changes in tissue structure and function. These alterations in tissue and organ function or gene expression patterns often persist from intrauterine life to adulthood and throughout the lifespan, resulting in a cascade of changes in the adult offspring.^[^ ^73^, ^74^ ^]^ In this study, we observed suppressed expression of InsR, p‐Akt‐S473/Akt, and Glut2 in the liver of PCE offspring at the intrauterine stage (GD20), followed by enhanced expression at the early postnatal stage (PW24). These changes exhibited a clear GC‐dependent and inverse relationship. Furthermore, in vitro studies confirmed that glucocorticoid regulation (rather than caffeine) had similar effects to those observed in vivo, suggesting the involvement of GC (rather than caffeine) in programming the hepatic insulin signaling pathway and glucose uptake function in PCE offspring rats. Previous studies have also demonstrated the involvement of GC in programming developmental changes and susceptibility to various diseases in multiple organs of PCE offspring, such as the adrenal glands, liver, kidneys, and ovaries.^[^ ^41^, ^75^, ^76^, ^77^, ^78^, ^79^, ^80^ ^]^ Additionally, high levels of intrauterine GC exposure have been shown to be a common cause of developmental abnormalities and disease susceptibility in offspring, resulting from various adverse factors during pregnancy, such as malnutrition, unhealthy habits, stress, and infections.^[^ ^71^, ^81^, ^82^ ^]^ This intrauterine GC‐rich environment may arise from significant stimulation of the maternal hypothalamic‐pituitary‐adrenal axis in response to environmental challenges, leading to the transmission of GC secretion signals and/or impaired placental GC barrier function (e.g., 11β‐HSD2 and P‐gp).^[^ ^81^, ^83^ ^]^ Intrauterine exposure to high GC levels can program adrenal development and function in offspring, resulting in postnatal adrenal hypofunction and low GC levels.^[^ ^75^, ^76^, ^77^, ^82^ ^]^ Therefore, these findings partially explained why caffeine use during pregnancy, due to its GC programming effect, can have a series of deleterious effects rather than beneficial effects observed in adult individuals.

GC primarily exerts an important regulatory effect on development, growth, and metabolism by binding to its receptor, GR, which activates GR translocation into the nucleus and can influence downstream target gene expression through the coordination of multiple epigenetic regulators.^[^ ^84^, ^85^ ^]^ miRNAs, as a form of epigenetic regulation, have been shown to be closely related to the occurrence of fetal IR. For example, maternal dietary restriction during pregnancy upregulates miR‐29a in IUGR offspring, leading to the inhibition of Glut4 expression in skeletal muscle, which partially contributes to decreased insulin‐dependent glucose uptake;^[^ ^86^ ^]^ Maternal chromium restriction induces specific miRNAs (miR‐327, miR‐466f‐3p, and miR‐223‐3p) that affect liver insulin signaling pathway in adult offspring mice, leading to IR.^[^ ^87^ ^]^ In this study, we identified differentially expressed miR‐1224 in the liver of PCE offspring rats, which targeted the regulation of InsR and was modulated by GC mediated by GR. Specifically, consistent changes in GC levels, GR expression, and miR‐1224 expression were observed in PCE offspring rats during the prenatal and early postnatal stages. In vitro studies further demonstrated that high concentrations of GC treatment in BMSCs‐differentiated hepatocyte‐like cells induced GR and miR‐1224 expression, resulting in the inhibition of the insulin signaling pathway and glucose uptake function. GR knockdown or miR‐1224 reversed the inhibitory effects of corticosterone on the insulin signaling pathway and glucose uptake function. Additionally, in HepG2 cells, decreasing GC concentrations were associated with decreased GR and miR‐1224 expression, accompanied by enhanced insulin signaling pathway and glucose uptake function. These data suggested that glucocorticoids program the hepatic insulin signaling pathway and glucose uptake function in PCE offspring during the prenatal and early postnatal stages through the GR/miR‐1224.

### 3.3 Accumulation of Ages Promotes the Onset of Late‐Stage IR in PCE Offspring

In this study, PCE offspring rats exhibited IR and abnormal glucose metabolism characterized by hyperglycemia and hyperinsulinemia in the late stage (after PW36). During this period, despite the decreased or downward trend in blood corticosterone levels and liver GR/miR‐1224 expression in PCE offspring, insulin signaling pathways and glucose uptake function were significantly reduced, in contrast to the changes observed in the early stage. These data suggested the involvement of other mechanisms in the regulation of insulin signaling pathways and glucose uptake function in the late‐stage life of PCE offspring. A recent study showed a negative correlation between birth weight (adjusted for gestational age) and levels of skin autofluorescence AGEs, with an average skin autofluorescence AGEs decrease of 0.056 (unit) per 1 kg increase in birth weight (adjusted for gestational age).^[^ ^23^ ^]^ Our previous research demonstrated that overexpression of Glut1 increases glucose uptake and leads to the accumulation of AGEs in the articular cartilage of PCE offspring rats.^[^ ^47^ ^]^ In this study, we also observed enhanced insulin sensitivity in the early stage of PCE offspring rats and significantly increased expression of the liver's main glucose uptake transporter, Glut2. Therefore, these early changes may accelerate the accumulation of AGEs in PCE offspring. Indeed, blood and liver AGE levels were observed to be significantly higher in the late stage compared to the control group in female PCE offspring. Accumulated AGEs have been shown to bind to their receptor, RAGE, in various cells and tissues, promoting the synthesis and release of multiple inflammatory factors such as IL‐6 and TNF‐α, triggering a series of oxidative stress and inflammatory cascades, thereby inhibiting insulin signaling pathways and inducing IR.^[^ ^62^, ^63^, ^88^, ^89^ ^]^ In this study, we found that the expression of RAGE and the levels of related inflammatory factors, IL‐6 and TNF‐α, were significantly increased in the late stage (PW40 and PW52) of PCE offspring rats. Furthermore, correlation analysis revealed a significant positive correlation between serum AGEs levels and the HOMA‐IR index in the PCE group at PW40 and PW52. In vitro experiments further confirmed that exogenous AGEs treatment significantly inhibits the increase in insulin signaling pathway and glucose uptake function induced by corticosterone below physiological concentration. And the above effects caused by AGEs were significantly blocked by RAGE antagonist FPS‐ZM1. These findings suggested that enhanced insulin sensitivity in the early stage of PCE offspring can lead to the accumulation of hepatic AGEs, which can further induce an inflammatory response through the AGE‐RAGE signaling, thereby negatively regulating insulin signaling pathways and glucose uptake function, ultimately resulting in the development of late‐stage hyperglycemia and hyperinsulinemia‐associated IR.

Hyperglycemia and the development of IR are also frequently associated with excessive gluconeogenesis. In this study, we also observed sustained upregulated expression of hepatic gluconeogenic enzymes (*Pck1* and *G6Pc*) in the PCE offspring from intrauterine to postnatal stages, which is highly consistent with the common phenomenon from a series of previous studies on rodent offspring suffering suboptimal intrauterine environments (reviewed by Christoforou et al^[^ ^90^ ^]^). Therefore, the long‐term programmed enhancement of hepatic gluconeogenesis in PCE offspring may also contribute to the development of IR. Interestingly, we observed that PCE offspring rats subjected to chronic stress^[^ ^91^ ^]^ for 14 days [ice water swimming (5–7 °C), 5 min per day from PW51 to PW52] exhibited a decrease in blood insulin level and a further increase (/worsening) in serum glucose level in PW52 (Figure S10, Supporting Information), indicating the susceptibility of PCE offspring pancreatic β‐cells to adverse stimuli and a compromised compensatory response. This result also highlighted the need to investigate the pancreatic β‐cell function in PCE offspring.

### 3.4 Onset of IR in PCE Offspring Under Normal Conditions May Precede Lipid Metabolism‐Related Disorders

Our previous studies have shown that PCE offspring rats can NAFLD under high‐fat diet or chronic stress conditions, and have demonstrated its intrauterine origin mechanism.^[^ ^46^, ^48^ ^]^ However, under normal diet feeding, the occurrence of NAFLD was not observed in PCE offspring rats at PW12, PW24, and PW40;^[^ ^41^, ^46^, ^48^, ^92^ ^]^ and even at PW52, as indicated by the serum triglyceride levels as well as histopathology and lipid metabolism‐related gene expression changes in liver (Figure S11, Supporting Information). Interestingly, under normal diet feeding, impaired glucose tolerance and insulin sensitivity were observed in PCE offspring rats at PW36, and noticeable IR was observed starting from PW40. These data suggested that under normal conditions, the onset of IR in PCE offspring may precede lipid metabolism‐related disorders such as NAFLD.

### 3.5 Clinical Recommendations

Unlike the beneficial effects of caffeine intake by adults on various adult diseases,^[^ ^29^, ^30^ ^]^ strong evidence from our and other laboratory studies has revealed that maternal caffeine intake during pregnancy can cause multiple adverse effects in the offspring, even at previously considered “safe” doses.^[^ ^31^, ^32^, ^33^, ^34^, ^35^, ^36^, ^37^, ^38^, ^39^, ^40^, ^41^, ^42^, ^43^, ^44^ ^]^ These studies raise concern that there may be no one absolute “safe” threshold of caffeine consumption during pregnancy. Beyond the F1 offspring, disorders induced by maternal caffeine exposure may also be transferred to the second and/or third generations.^[^ ^78^, ^93^, ^94^, ^95^, ^96^ ^]^ Although the World Health Organization and European Food Safety Authority recommend that daily caffeine consumption remain below 200–300 mg as a safe dosage for pregnant health,^[^ ^97^, ^98^ ^]^ we strongly recommend that pregnant women avoid caffeine‐containing foods and beverages as much as possible, for the sake of the health of their children and grandchildren. Mechanistically, the adverse effects of caffeine on F1 offspring could be due to early embryo caffeine exposure via oviductal or uterine fluid,^[^ ^99^ ^]^ or during later exposure that bypasses the blood‐placenta barrier. In addition to the direct effect of caffeine exposure, caffeine intake during pregnancy can cause an increase in maternal glucocorticoids.^[^ ^100^ ^]^ This study, as well as several of our previous studies^[^ ^78^, ^94^, ^95^, ^96^ ^]^ have shown that a fetus exposed to such an environment can result in long‐term programming of glucocorticoids, which could increase susceptibility to multiple diseases in F1 offspring, even the second and/or third generations. Maternal glucocorticoid levels in the fetus are mainly regulated by the placental glucocorticoid barrier, including 11β‐hydroxysteroid dehydrogenase type 2 (11β‐HSD2) and P‐glycoprotein (P‐gp). The 11β‐HSD2 can reduce glucocorticoids from entering fetal circulation by inactivating glucocorticoids.^[^ ^101^ ^]^ P‐gp in the placental trophoblasts can use ATP to pump glucocorticoids back to the maternal circulation.^[^ ^102^ ^]^ Our previous study confirmed that P‐gp inducer (sodium ferulate) can reverse the effect of caffeine on the fetal/placental weights.^[^ ^38^ ^]^ This implied that intervention in the placental glucocorticoid barrier has the potential to prevent and treat offspring IUGR and related disorders induced by adverse factors such as caffeine through maternal high glucocorticoid.

### 3.6 Limitations

IUGR is a result of multiple factors, and the mother, placenta, and fetus have been identified as three major parts of risk factors for IUGR.^[^ ^103^, ^104^ ^]^ Research has shown that fetal over‐exposure to maternal glucocorticoids is one of the important causes of IUGR occurrence.^[^ ^105^, ^106^ ^]^ This study established an IUGR rat model using caffeine as a chronic stressor. The characteristic of this model is the presence of high levels of glucocorticoids in the mother, leading to excessive exposure of the fetus to glucocorticoids, which has also been confirmed in previous studies.^[^ ^38^, ^59^ ^]^ Under this model, we elucidated the changes in insulin sensitivity of IUGR female offspring before and after birth, and demonstrated that this was associated with intrauterine exposure to high corticosteroid levels. This suggests that the findings of this study may be applicable to IUGR female offspring under various factors that can cause abnormal elevation of glucocorticoids. In addition, it should be emphasized that the IUGR model with GC over‐exposure may not represent all IUGR subtypes. There are some other IUGR models (e.g., utero‐placental deficiency) that lead to altered glucose tolerance and insulin sensitivity in offspring without GC‐overexposure.^[^ ^107^ ^]^

Both epidemiological surveys and animal experiments identify that there is a sex difference in chronic adult‐onset diseases with fetal origin.^[^ ^108^ ^]^ Our previous studies also indicated that the programming effect of PCE on glucose homeostasis and tolerance in offspring rats is characterized by sex difference: changes in females are more significant than those in their male counterparts.^[^ ^42^, ^109^ ^]^ In this study, despite the dynamic changes in insulin sensitivity of female IUGR offspring rats induced by PCE at different stages after birth being demonstrated, there is a limitation in the lack of male data. A more comprehensive study comprising male offspring deserves to be conducted in the future.

## 4 Conclusion

For the first time, we observed age‐dependent changes in insulin sensitivity in female PCE offspring with IUGR after birth, characterized by a shift from sensitive to resistant. The hepatic GR/miR1224 programming enhanced insulin signaling in the early stage, which contributed to improved insulin sensitivity. However, the consequent increased glucose uptake induced excessive AGEs accumulation in the liver, conversely, inhibited insulin signaling via RAGE, which ultimately led to IR occurrence.

## 5 Experimental Section

### Chemicals and Reagents

Details of the main chemicals and reagents are shown in Table S1 (Supporting Information). All other chemicals were of analytical grade and were commercially available.

### Animals and Treatment

Specific pathogen‐free Wistar rats (male body weight: 230 ± 20 g, female body weight: 200 ± 20 g) were provided by Hubei Provincial Academy of Preventive Medicine (Wuhan, Hubei, China). Rats were kept in a controlled environment (temperature of 23 ± 2 °C, relative humidity of 50 ± 10%, and a 12 h light/dark cycle) with free access to water and food. All animal experimental procedures were reviewed and approved by the Institutional Animal Care and Use Committee (IACUC) of the Center for Animal Experiment of Wuhan University (IACUC NO. WP20210476).

The PCE‐induced IUGR rat model was established based on our previous research.^[^ ^38^, ^40^, ^41^, ^48^ ^]^ The animal experimental protocol is shown in Figure S1 (Supporting Information). In brief, pregnant rats were divided into a control group (Control), low‐dose caffeine group [30 mg(kg·d)^−1^; PCE(L)], and high‐dose caffeine group [120 mg(kg·d)^−1^; PCE(H)]. From gestational day (GD) 9 to 20, pregnant rats were orally administered caffeine [30 or 120 mg(kg·d)^−1^] or an equivalent volume of vehicle solution.

For prenatal experiments, overnight fasting was implemented, and pregnant rats were anesthetized with 2% isoflurane and euthanized 2 h after the last dose on GD20. Fetuses with a litter size ranging from 8 to 14 were used for the experiments. After determining the gender of the fetal pups, they were weighed. Subsequently, the fetal blood and liver tissues were collected after euthanizing the rats under 2% isoflurane anesthesia and stored at −80 °C for further analysis.

For postnatal experiments, some control group and PCE(H) group pregnant rats underwent natural delivery. To ensure nutrition, the litter size was adjusted to 12 pups (6♀+ 6♂) per dam on the first day after birth (postnatal day 1, PD1). At postnatal week 4 (PW4), the offspring rats were weaned and fed a standard laboratory diet until the time of sampling. Specifically, the following procedures were performed: ① One randomly selected female offspring rat from each litter was used to assess dynamic changes in insulin sensitivity and confirm the occurrence of IR (n = 12/group). Blood samples were obtained by tail tipping at PW8, PW16, PW24, PW32, PW40, and PW52 to collect serum. At PW52, intraperitoneal glucose tolerance test (IPGTT) and intraperitoneal insulin tolerance test (IPITT) were conducted. Afterward, the rats were euthanized under 2% isoflurane anesthesia, and blood and liver tissues were collected and stored at ˗80 °C for further analysis. ② Additionally, two randomly selected female offspring rats from each litter were subjected to IPGTT and IPITT at PW24 and PW36, respectively (n = 12/group). After euthanasia under 2% isoflurane anesthesia, blood and liver tissues were collected and stored at ˗80 °C for further analysis. The remaining female and male offspring rats from each litter were used for other experimental investigations.

### IPGTT and IPITT

The IPGTT and IPITT were conducted as described previously.^[^ ^42^, ^49^, ^50^ ^]^ In brief, for IPGTT, all rats were fasted for 12 h and then intraperitoneally injected with glucose (2 g/kg body weight). Blood samples were collected from the tail vein at 0, 15, 30, 60, and 120 min after injection, and blood glucose levels were measured using a Roche Accu‐Chek glucometer. For IPITT, rats were fasted for 6 h and then intraperitoneally injected with insulin (0.75 Ukg^−1^). Blood samples were collected from the tail vein at 0, 15, 30, 60, and 120 min after injection, and blood glucose levels were measured using a glucometer. To eliminate fasting blood glucose differences caused by individual states, the blood glucose levels at each time point of IPGTT and IPITT were standardized to the 0‐min value to obtain relative values. The change curves were plotted, and the area under the curve (AUC) was calculated. The AUC was calculated using the trapezoidal rule as follows:

(1) $$\begin{array}{ccc} \mathrm{AUC} & = & \frac{\left(C_{0}+C_{15}\right)}{2}\times Time_{0-15}+\frac{\left(C_{15}+C_{30}\right)}{2}\times Time_{15-30}\\ & & +\frac{\left(C_{30}+C_{60}\right)}{2}\times Time_{30-60}+\frac{\left(C_{60}+C_{120}\right)}{2}\times Time_{60-120} \end{array}$$

(2) $$C_{X}:\mathrm{Blood}\;\mathrm{glucose}\;\left(\mathrm{mM}\right);\mathrm{Time}:\mathrm{Interval}\;\left(\min\right)$$

### Biochemical Assay and Enzyme Linked Immunosorbent Assay (ELISA)

After allowing whole blood to stand at 4 °C for 15 min, serum was obtained by centrifugation at 3500 rpm for 15 min and stored at ˗80 °C until analysis. Serum glucose, insulin, corticosterone, AGEs, tumor necrosis factor α (TNF‐α), and nterleukin 6 (IL‐6) levels were determined using their respective commercial assay kits according to the manufacturer's instructions. IR was evaluated by the insulin resistance index (IRI) calculated using the homeostasis model assessment of insulin resistance (HOMA‐IR) method. The calculation formula for HOMA‐IR is as follows:

(3) $$\mathrm{HOMA}-\mathrm{IR}=\frac{\mathrm{Fasting}\;\mathrm{insulin}\;\left(\mu \mathrm{IU}/\mathrm{mL}\right)\times \mathrm{Fasting}\;\mathrm{glucose}\;\left(\mathrm{mM}\right)}{22.5}$$

### Immunohistochemical (IHC) Analysis

The IHC analysis was conducted as described previously.^[^ ^51^ ^]^ In brief, randomly selected liver sections from three offspring in each group (from different litters) were collected and fixed in 4% paraformaldehyde, followed by embedding in paraffin. The paraffin‐embedded liver tissues were then cut into 4‐µm thick sections for IHC staining. The liver tissue sections were subjected to routine dewaxing and antigen retrieval by heating in a microwave for 10 min using citrate buffer (0.01 M, pH 6.0). Subsequently, the sections were treated with distilled water containing 0.3% hydrogen peroxide for 20 min to block endogenous peroxidase activity. After washing with phosphate buffered saline (PBS) three times, the sections were blocked with serum for 1 h, followed by incubation with a mouse monoclonal antibody against AGEs (dilution 1:200) for 2 h. After washing with PBS three times, the sections were incubated with biotinylated goat anti‐mouse IgG for 45 min, followed by incubation with a streptavidin‐peroxidase complex prepared according to the manufacturer's instructions for 45 min. Finally, the peroxidase reaction was visualized using 3,3‐diaminobenzidine as the substrate. After completion of the color development, the sections were counterstained with Harris hematoxylin for 5 s, rinsed in running tap water for 20 min, dehydrated using an ethanol series, cleared with xylene, and mounted with Canada balsam. Photomicrographs were captured and the staining intensity (i.e., average optical density) was assessed using NIS Elements Br 4.20 software (Nikon, USA).

### Cell Culture and Treatment

The process of culturing and differentiating bone marrow mesenchymal stem cells (BMSCs) into hepatocyte‐like cells was performed as described previously.^[^ ^52^ ^]^ Briefly, BMSCs were obtained from 3‐week‐old female rats and cultured in Minimum Essential Medium (MEM) α containing 10% fetal bovine serum, 100 mgmL^−1^ streptomycin, and 100 UmL^−1^ penicillin in a humidified atmosphere of 5% CO_2_ at 37 °C. When the cells reached 80% confluence in six‐well plates, they were differentiated into hepatocyte‐like cells using Improved MEM (IMEM) supplemented with 1% fetal bovine serum, 100 mg/mL streptomycin, 100 U/mL penicillin, 20 ngmL^−1^ hepatocyte growth factor, 2 ngmL^−1^ epidermal growth factor, 0.1 µM dexamethasone, and 50 mgmL^−1^ insulin‐transferrin‐selenium for 14 d. After differentiation, the BMSC‐derived hepatocyte‐like cells were treated with different concentrations of caffeine (0, 0.1, 1, 10 µM) or corticosterone (0, 300, 600, 1200 nM) for 48 h, and the cells were collected for subsequent analyses.

HepG2 cells were cultured in Dulbecco's Modified Eagle Medium (DMEM) containing 10% fetal bovine serum, 100 UmL^−1^ penicillin, and 100 µgmL^−1^ streptomycin in a humidified atmosphere of 5% CO_2_ at 37 °C. After seeding HepG2 cells in six‐well plates, they were treated with different concentrations of corticosterone (500, 200, 125, 62.5 nM) or AGE‐bovine serum albumin (AGE‐BSA) (0, 50, 100, 200 µgmL^−1^) for 48 h, and the cells were collected for subsequent analyses. To investigate the effect of AGEs on the action of low‐dose corticosterone, the following treatment groups were performed: corticosterone (500 nM) + BSA (200 µgmL^−1^), corticosterone (62.5 nM) + BSA (200 µg/mL), corticosterone (62.5 nM) + AGE‐BSA (200 µgmL^−1^), and corticosterone (500 nM) + AGE‐BSA (200 µg/mL), and the cells were collected for subsequent analyses after 48 h treatment.

### Transfection and Dual‐Luciferase Reporter Gene Assays

siRNA was chemically synthesized by Suzhou Genepharma Co., Ltd. (Suzhou, Jiangsu, China). According to the manufacturer's instructions, glucocorticoid receptor (GR) siRNA (sequence: sense‐CCAGAGAUGUU AGCUGAAATT, antisense‐UUUCAGCUAACAUCUCUGGTT) was transfected into differentiated BMSCs using Lipofectamine 3000, followed by a 24‐h incubation. After transfection, the transfected cells were subjected to drug treatment.

A luciferase assay was performed to demonstrate the direct binding of miR‐1224 to the predicted binding site in the 3′‐UTR of InsR mRNA. Recombinant plasmids containing the miR‐223 binding site in the InsR‐3′UTR were constructed as InsR‐WT, and a vector with a seed sequence lacking the miR‐1224 binding site was used as InsR‐MUT. HepG2 cells were co‐transfected with InsR‐WT or InsR‐MUT constructs along with miR‐223 mimics or control mimics using Lipofectamine 3000, as per the manufacturer's instructions. The vectors expressed firefly luciferase and Renilla luciferase. After 48 h, the relative luciferase activity was measured using a dual‐luciferase reporter gene assay kit, with the ratio of firefly to Renilla luciferase activity representing the relative luciferase activity.

### 2‐NBDG Uptake Assays

BMSCs or HepG2 cells were seeded in a 96‐well plate and subjected to drug treatment. Following treatment, each well was replenished with 100 µL of glucose‐free, serum‐free culture medium containing the respective drug and incubated for 3 h under starvation conditions. The cells were then washed twice with PBS and incubated with 100 µL of glucose‐free, serum‐free culture medium containing 100 mM 2‐Deoxy‐2‐ [(7‐nitro‐2,1,3‐benzoxadiazol‐4‐yl) amino]‐D‐glucose (2‐NBDG) for 30 min. Subsequently, the culture medium was removed, and the wells were washed twice with PBS before adding 100 µL of PBS. Fluorescence intensity of 2‐NBDG was measured using a multifunctional microplate reader with excitation wavelength set at 470 nm and emission wavelength set at 540 nm.

### MicroRNA‐seq Assays

Liver tissue samples from three litters of GD20 female fetal rats were collected and pooled as a single sample for MicroRNA‐seq analysis. miRNA library preparation, high‐throughput sequencing, and miRNA‐seq data analysis were performed by Novogene Bioinformatics Technology Co., Ltd (Beijing, China). Briefly, the miRNA library was generated using NEB Next Multiplex Small RNA Library Prep Set for Illumina (NEB E7300L). Subsequently, library quality was assessed on the Agilent 5400 system (Agilent, USA) and quantified by QPCR (1.5 nM). The Qualified libraries were pooled and sequenced on Illumina platforms with SE50 strategy, according to effective library concentration and data amount required. miRNA expression levels were estimated by TPM (transcript per million) through the following criteria.^[^ ^53^ ^]^ Normalization formula: Normalized expression = mapped readcount/Total reads*1000000. For the samples without biological replicates, Differential expression analysis of two samples was performed using the DEGseq (2010) R package. *P*<0.01 and |log2(fold change)| > 1 was set as the threshold for significantly differential expression by default.

### Reverse Transcription‐Quantitative Real‐Time Polymerase Chain Reaction (RT‐qPCR) Assays

RT‐qPCR was performed as described previously.^[^ ^50^, ^51^, ^54^, ^55^, ^56^ ^]^ Total RNA was isolated from tissues and cells using TRIzol reagent. The yield and purity of the isolated RNA were determined using a NanoDrop 2000 spectrophotometer (Thermo Scientific, Wilmington, DE, USA). The samples with A260/A280 ratio ranging from 1.8 to 2.05 were used for cDNA synthesis. For miRNA analysis, cDNA was synthesized using the miScript II RT kit. For mRNA detection, 1 µg of total RNA was reverse transcribed into cDNA using the HiScript III RT SuperMix for qPCR (+gDNA wiper), following the manufacturer's instructions. The cDNA was diluted 1:10 with nuclease‐free water as the RT‐qPCR template. The miScript Green PCR kit and Cham Q Universal SYBR qPCR Master Mix were used for qPCR detection on the QuantStudio 5 Real‐Time PCR System (Thermo Scientific, Waltham, MA, USA). The primer sequences for the genes are listed in Table S2 (Supporting Information). The expression levels of mRNA and miRNAs were calculated relative to the endogenous control genes, β‐actin and U6 small nuclear RNA (RNU6), respectively, using the 2^−ΔΔCt^ method.

### Western Blot (WB) Analysis

WB analysis was performed as described previously.^[^ ^50^, ^54^, ^57^ ^]^ Total protein was extracted from tissues or cells using commercially available kits following the manufacturer's instructions. Protein concentration was determined using the bicinchoninic acid (BCA) method. Subsequently, the samples were separated on a 10% sodium dodecyl sulfate‐polyacrylamide gel and transferred to a polyvinylidene fluoride membrane. After blocking with 5% nonfat milk in 1× TBS + Tween (TBST) at room temperature for 2 h, the membrane was incubated overnight at 4 °C with diluted antibodies against InsR (1:1000 dilution), Akt (1:1000 dilution), p‐Akt‐S473 (1:1000 dilution), Glut2 (1:1000 dilution), and β‐actin (1: 5000 dilution), respectively. After washing the membrane with TBST three times, it was incubated with secondary antibodies conjugated with horseradish peroxidase for 2 h. Specific target protein bands were visualized using an ECL kit, and further semi‐quantitative analysis was performed using Image Pro Plus 6.0 (Media Cybernetics, MD, USA).

### Statistical Analyses

All data were analyzed using IBM SPSS Statistics 20 (SPSS Science Inc., Chicago, IL, USA) and Graphpad Prism 9.0 (GraphPad Software, La Jolla, CA, USA). Results were presented as Mean ± standard error of mean (S.E.M.). Statistical analyses for comparisons of test groups with the control group were conducted using appropriate tests, based on the nature of the data set to be analyzed. After checking normality and homogeneity of variance, a two‐tailed unpaired Student's t‐test was used to compare two groups with normally distributed data and equal variances, while a two‐tailed unpaired Student's t‐test with Welch's correction was used to compare two groups with normally distributed data and unequal variances. One‐way analysis of variance (ANOVA) was used to compare multiple groups with normally distributed data and equal variances, while the One‐way Brown‐Forsythe ANOVA test was used to compare multiple groups with normally distributed data and unequal variances. The repeated measures data were analyzed by using Two‐way ANOVA for repeated measures followed by a Bonferroni post‐hoc test. The between‐subject factor is group (i.e., PCE versus control), and the within‐subject (repeated) factor is time. Detailed statistical analysis information can also be found in the figure legend. When P<0.05 was designated as a statistical difference.

## Conflict of Interest

The authors declare no conflict of interest.

## Author Contributions

Y. D. and X. G. contributed equally to this work. H.W. and H.K. secured funding. H. W. and H. K. conceived and designed the experiments. X.G. performed experiments. P.Y. and D.Z. analyzed the data. H.K. and Y.D. analyzed the data and wrote the manuscript. All authors contributed to the discussion, critical revision, and approved the final manuscript.

## Supporting information



Supporting Information

## Data Availability

The data that support the findings of this study are available from the corresponding author upon reasonable request.
