# Supplementary material for: Mechanisms and Pathophysiological Significance of Insulin Resistance in Offspring With Intrauterine Growth Restriction Mediated by Hepatic GR/miR‐1224 Programming
Source: Adv Sci (Weinh). 2025 Sep 3;12(44):e10277. doi: 10.1002/advs.202510277 (PMC12667461; doi:10.1002/advs.202510277)
Supplement: Supplementary file 1 — Supporting Information [file ADVS-12-e10277-s001.docx]

**Supplementary information**

**Mechanisms and Pathophysiological Significance of Insulin Resistance in Offspring with Intrauterine Growth** **Restriction Mediated by Hepatic GR/miR-1224 Programming**

Yongguo Dai *^a ,c, d^* ^#^, Xiaoling Guo *^a,c^*^,#^, Pengxia Yu *^a,c^*, Dingmei Zhang *^a,c^*, Hao Kou *^b,c,*^*, Hui Wang *^a,c,*^*

*^a^ Department of Pharmacology, School of Basic Medical Sciences, Wuhan University, Wuhan 430071, China;*

*^b^ Department of Pharmacy, Zhongnan Hospital of Wuhan University, Wuhan 430071, China;*

*^c^ Hubei Provincial Key Laboratory of Developmentally Originated Disease, Wuhan 430071, China.*

*^d^ Department of Pharmacy, National Clinical Research Center for Geriatric Disorders, Xiangya Hospital, Central South University, Changsha 410008, China.*

**E-mail and ORCID for each author:** Yongguo Dai (E-mail: [daiyongguo1995@csu.edu.cn](mailto:daiyongguo1995@csu.edu.cn); ORCID: <https://orcid.org/0000-0002-9023-5211>); Xiaoling Guo (E-mail: [guoxiaoling186@163.com](mailto:guoxiaoling186@163.com)); Pengxia Yu (E-mail: [yupengxiazy@163.com](mailto:yupengxiazy@163.com)); Dingmei Zhang (E-mail: [dingmeiz@qq.com](mailto:dingmeiz@qq.com)); Hao Kou (E-mail: [kouhao007@whu.edu.cn](mailto:kouhao007@whu.edu.cn)); Hui Wang (E-mail: [wanghui19@whu.edu.cn](mailto:wanghui19@whu.edu.cn); ORCID: <https://orcid.org/0000-0001-5300-8661>).

^#^ Yongguo Dai and Xiaoling Guo contributed equally to this study.

*** Corresponding authors:**

**Prof. Hui Wang**, Ph.D., Department of Pharmacology, School of Basic Medical Sciences, Wuhan University, Wuhan 430071, China. Tel: +86-13627232557, E-mail: [wanghui19@whu.edu.cn](mailto:wanghui19@whu.edu.cn); ORCID: <https://orcid.org/0000-0001-5300-8661>.

**Dr. Hao Kou**, Ph.D., Department of Pharmacy, Zhongnan Hospital of Wuhan University, Wuhan 430071, China. Tel: +86-13971609553, E-mail: [kouhao007@whu.edu.cn](mailto:kouhao007@whu.edu.cn).

**Supporting Figures:**


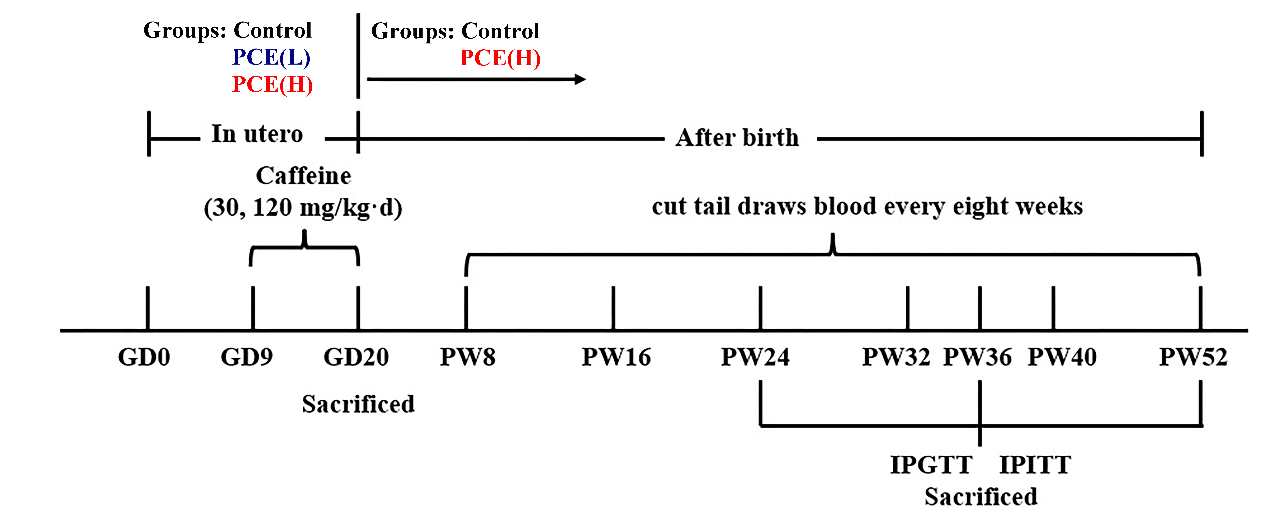


**Fig S1. Animal experimental procedures.**PCE(L), prenatal caffeine exposure [30 mg/(kg·d)]; PCE(H), prenatal caffeine exposure [120 mg/(kg·d)]; IPGTT, intraperitoneal glucose tolerance test; IPITT, intraperitoneal insulin tolerance test; GD, gestational day; PW, postnatal week.


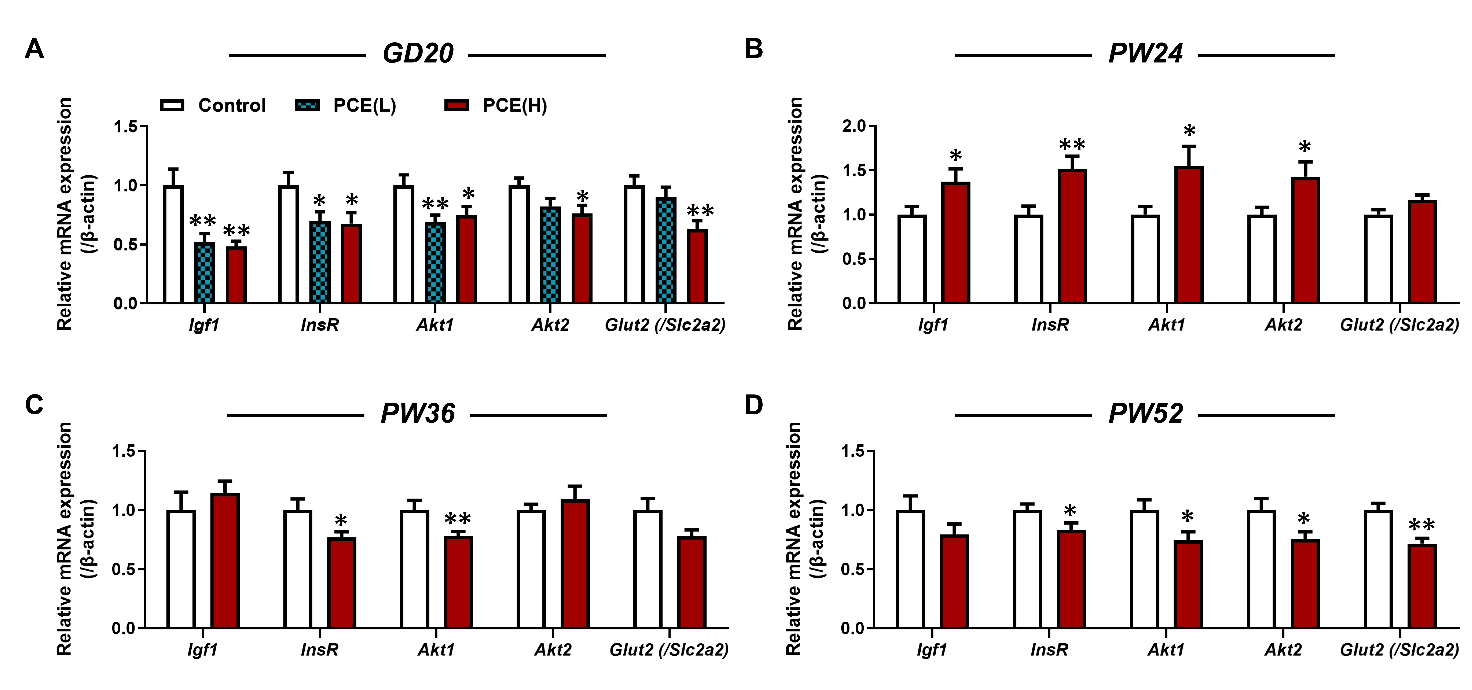


**Fig** **S2. Effects of PCE on the mRNA expression of hepatic IGF1, InsR, Akt1, Akt2 and Glut2 in different ages of female offspring rats.** (A) On GD20; (B) In PW24; (B) In PW36; (B) In PW52. The data are shown as Mean ± S.E.M., n=12. Two-tailed unpaired Student’s *t*-test (B-D), One-way ANOVA with Dunnett’s post-hoc test (A). ^*^*P<*0.05, ^**^*P<*0.01 *vs*. control. GD, gestational day; PW, postnatal week; *Igf1*, insulin-like growth factor 1; *InsR*, insulin receptor; *Akt1*, AKT serine/threonine kinase 1; *Akt2*, AKT serine/threonine kinase 2; *Glut2* (/*Slc2a2*), glucose transporter type 2; PCE(L), prenatal caffeine exposure [30 mg/(kg·d)]; PCE(H), prenatal caffeine exposure [120 mg/(kg·d)].


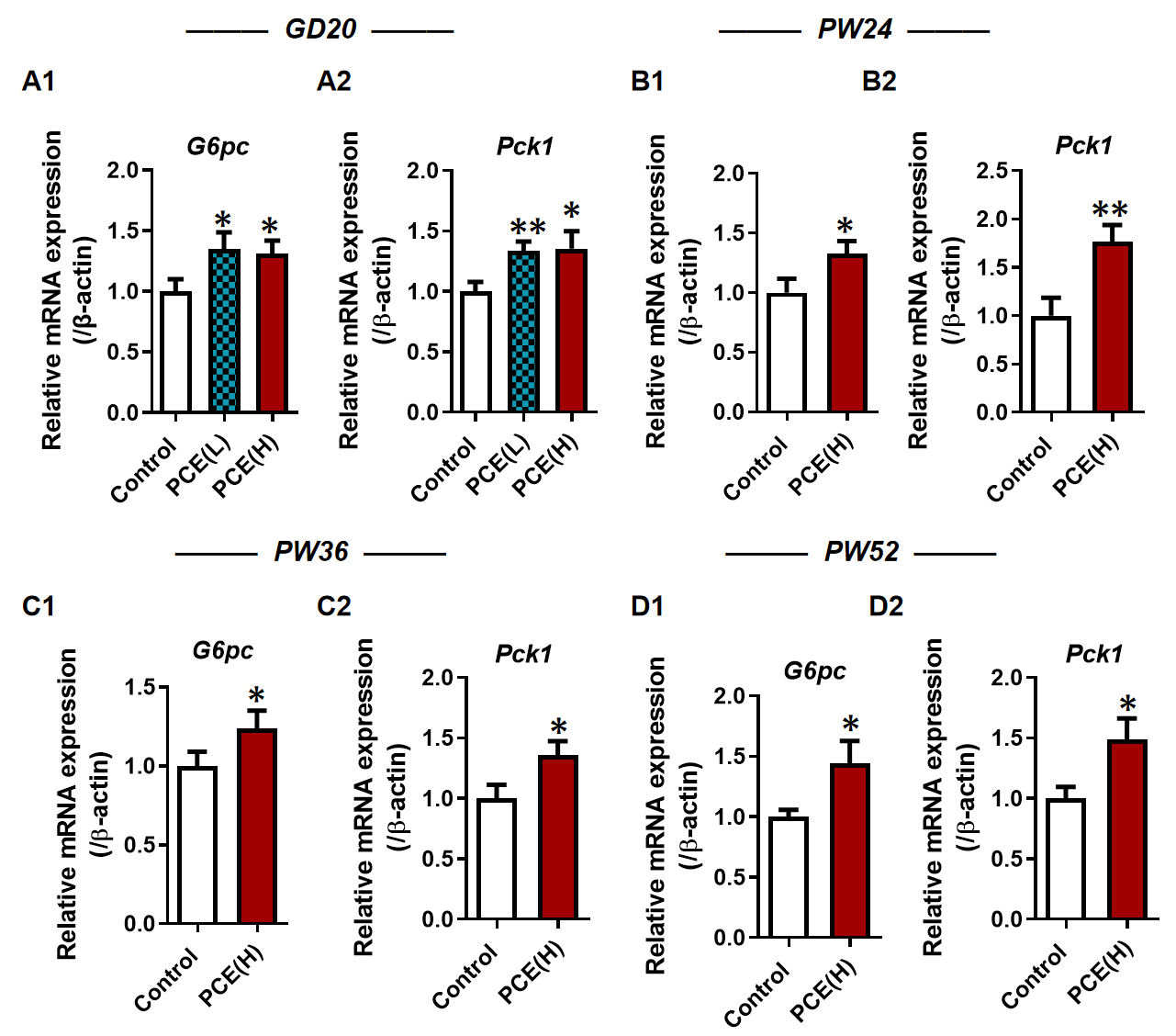


**Fig S3. Effects of PCE on** **the mRNA expression levels of the hepatic key gluconeogenesis enzymes in different ages of female offspring rats.** (A1-A2) The mRNA expression levels of *G6pc* and *Pck1* on GD20; (B1- B2) The mRNA expression levels of *G6pc* and *Pck1* in PW24; (C1-C2) The mRNA expression levels of *G6pc* and *Pck1* in PW36; (D1-D2) The mRNA expression levels of *G6pc* and *Pck1* in PW52. The data are shown as Mean ± S.E.M., n=12. Two-tailed unpaired Student’s *t*-test (B1, B2, C1, C2, D1, D2), One-way ANOVA with Dunnett’s post-hoc test (A1, A2). ^*^*P<*0.05, ^**^*P<*0.01 *vs*. control. *G6pc*, glucose-6-phosphatase; *Pck1*, phosphoenolpyruvate carboxykinase, GD, gestational day; PW, postnatal week; PCE(L), prenatal caffeine exposure [30 mg/(kg·d)]; PCE(H), prenatal caffeine exposure [120 mg/(kg·d)].


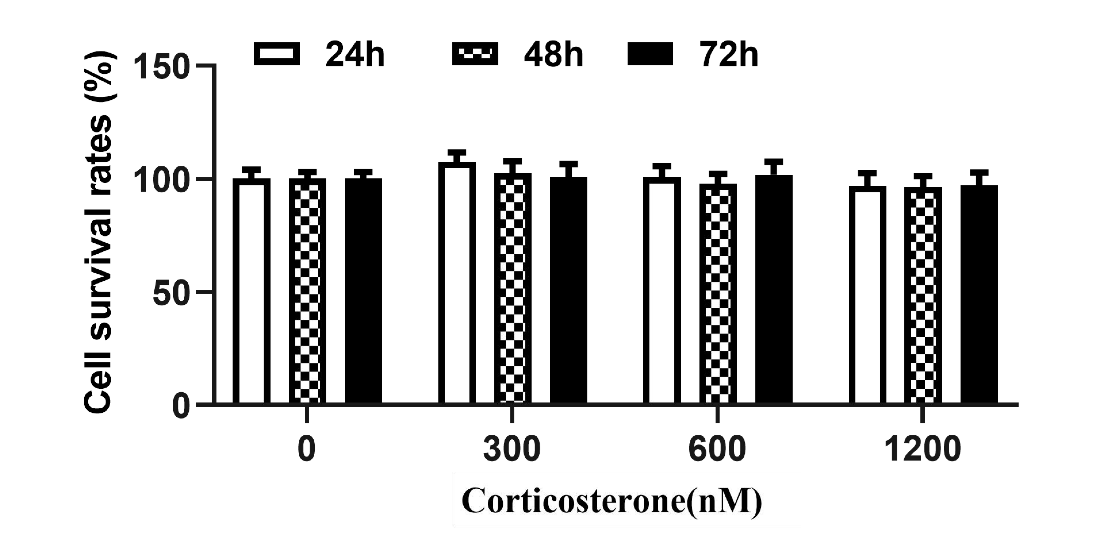


**Fig S4. Cell viability of hepatocyte-like cells differentiated from rat bone marrow mesenchymal stem cells (BMSCs) after treatment with high concentration of corticosterone.** The data are shown as Mean ± S.E.M., n=6. One-way ANOVA with Dunnett’s post-hoc test.


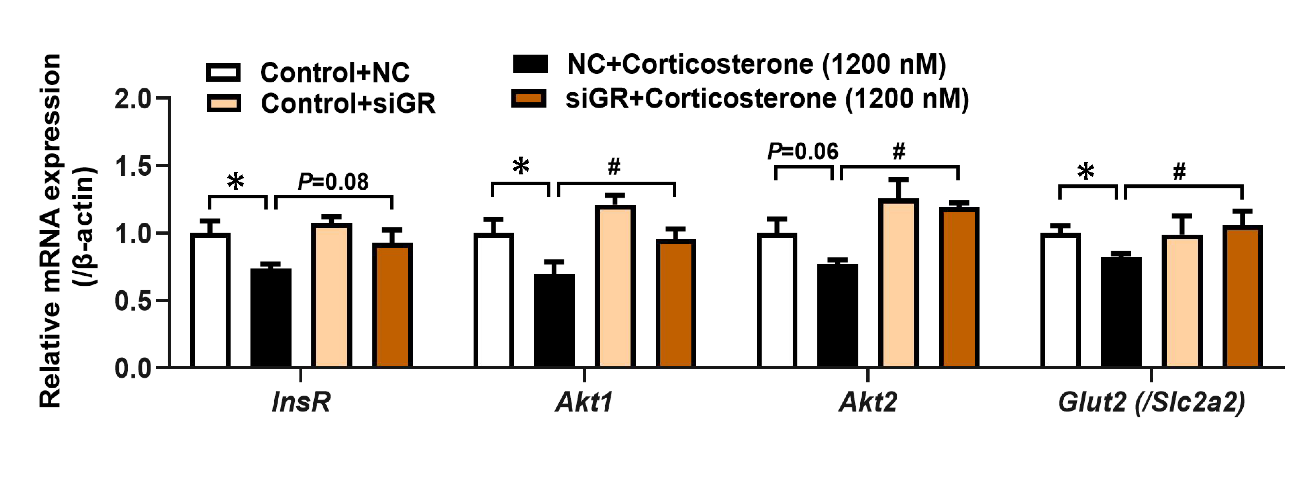


**Fig S5. The mRNA expression of *InsR*, *Akt1*, *Akt2*, *Glut2* (/*Slc2a2*) in hepatocyte-like cells differentiated from rat bone marrow mesenchymal stem cells (BMSCs) after treatment with siGR and/or high concentration of corticosterone.** The data are shown as Mean ± S.E.M., n=6. One-way ANOVA with Tukey’s post-hoc test. ^*^*P*<0.05 *vs*. control; *^#^P*<0.05 *vs.* corticosterone-treated group. BMSCs, bone marrow mesenchymal stem cells; *InsR*, insulin receptor; *Akt1*, AKT serine/threonine kinase 1; *Akt2*, AKT serine/threonine kinase 2; *Glut2* (/*Slc2a2*), glucose transporter type 2.


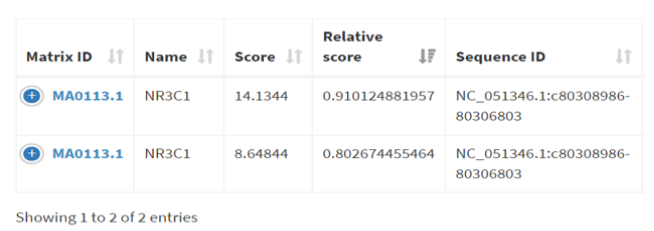


**Fig S6. Predictive analysis of the presence of GR(/NR3C1) binding sites in the promoter region of miR-1224 by JASPAR website (**[**https://jaspar.genereg.net/**](https://jaspar.genereg.net/)**).**


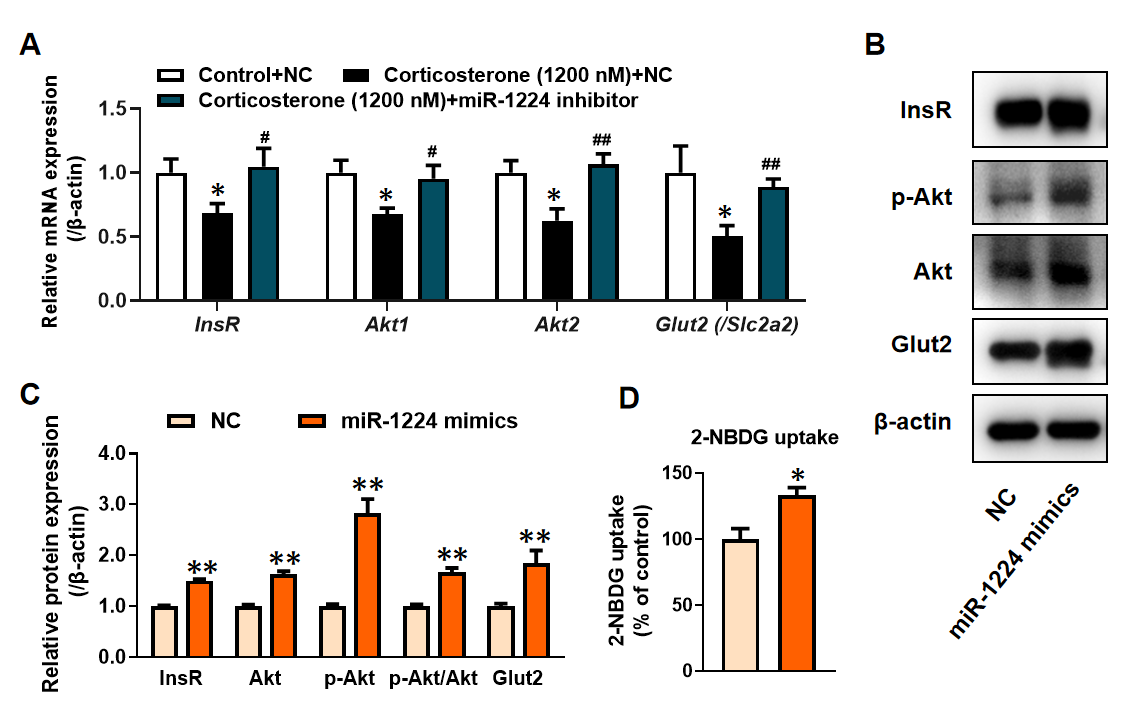


**Fig S7. The changes of insulin signaling pathway and glucose uptake function in hepatocyte-like cells differentiated from rat BMSCs after treatment with miR-1224 inhibitors or miR-1224** **mimics.** (A) The mRNA expression of *InsR*, *Akt1*, *Akt2*, *Glut2* (/*Slc2a2*) in hepatocyte-like cells differentiated from rat BMSCs after treatment with miR-1224 inhibitor and/or high concentration of corticosterone**;** (B, C) Protein expression of InsR, Akt, p-Akt-S473 and Glut2 in hepatocyte-like cells differentiated from rat BMSCs after treatment with miR-1224 mimics; (H) 2-NBDG uptake in hepatocyte-like cells differentiated from rat BMSCs after treatment with miR-1224 mimics**.** The data are shown as Mean ± S.E.M., n=3 for protein expression assay, n=6 for other detection. Two-tailed unpaired Student’s *t*-test (C, D), One-way ANOVA with Tukey’s post-hoc test (A). ^*^*P*<0.05, ^**^*P<*0.01 *vs*. control; *^#^P*<0.05, ^##^*P<*0.01 *vs.* corticosterone-treated group. BMSCs, bone marrow mesenchymal stem cells; InsR, insulin receptor; Glut2 (/Slc2a2), glucose transporter type 2; Akt, AKT serine/threonine kinase; p-Akt, phosphorylated Akt; 2-NBDG, 2-Deoxy-2-[(7-nitro-2,1,3-benzoxadiazol-4-yl) amino]-D-glucose.


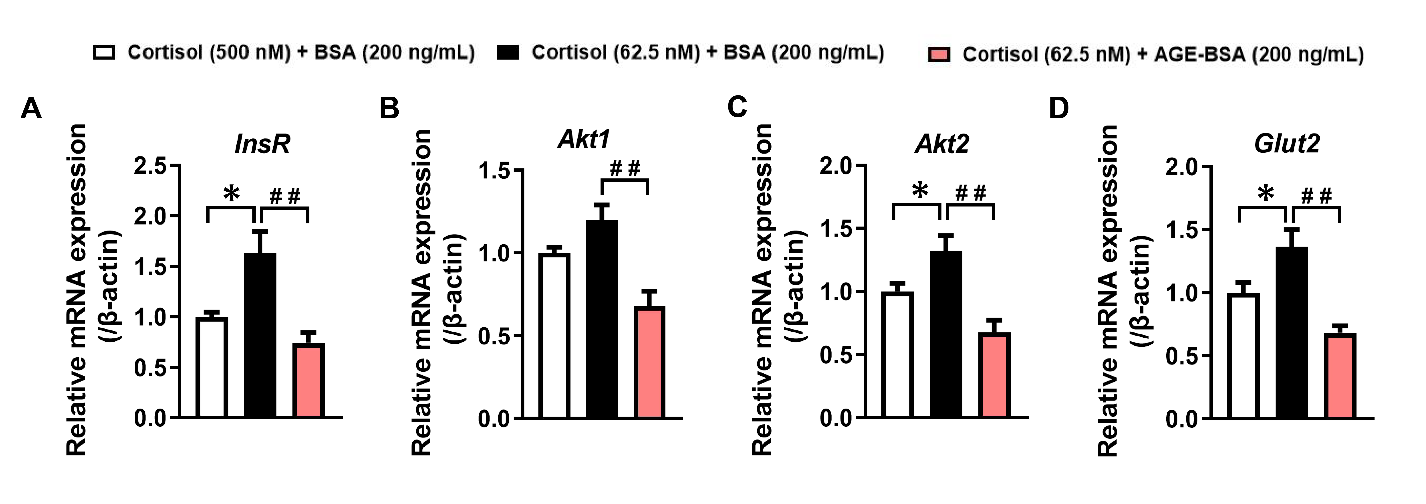


**Fig S8. The mRNA expression of *InsR*, *Akt1*, *Akt2*, *Glut2* (/*Slc2a2*) in HepG2 cells after treatment with AGE-BSA under lower than physiological concentrations of cortisol.** The data are shown as Mean ± S.E.M., n=6. One-way ANOVA with Tukey’s post-hoc test. ^*^*P*<0.05 *vs*. control; ^##^*P<*0.01 *vs.* cortisol (62.5 nM)-treated group. *InsR*, insulin receptor; *Akt1*, AKT serine/threonine kinase 1; *Akt2*, AKT serine/threonine kinase 2; *Glut2* (/*Slc2a2*), glucose transporter type 2.


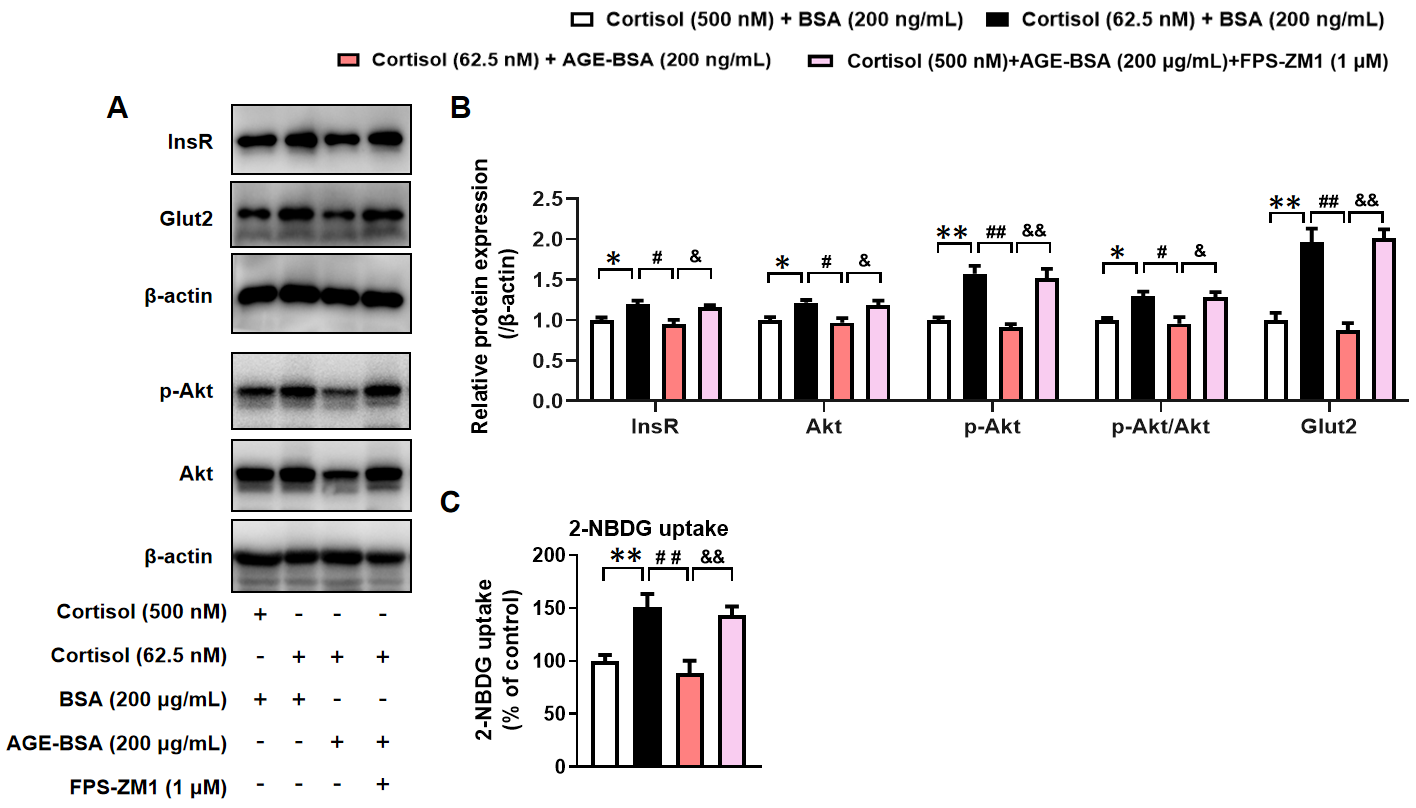


**Fig S9. RAGE antagonist FPS-ZM1 blocks the inhibition of AGEs on insulin signaling pathway and glucose uptake in HepG2 cells under lower than physiological concentrations of cortisol.** (A, B) Protein expression of hepatic InsR, Akt, p-Akt-S473 and Glut2 in HepG2 cells after treatment with AGE-BSA and FPS-ZM1 under lower than physiological concentrations of cortisol; (L) 2-NBDG uptake in HepG2 cells after treatment with AGE-BSA and FPS-ZM1 under lower than physiological concentrations of cortisol. The data are shown as Mean ± S.E.M., n=3 for protein expression assay, n=6 for 2-NBDG uptake. One-way ANOVA with Tukey’s post-hoc test. ^*^*P<*0.05, ^**^*P<*0.01 *vs*. control (BSA and 500 nM cortisol) group; *^#^P<*0.05, ^##^*P<*0.01 *vs*. cortisol (62.5 nM)-treated group; *^&^P<*0.05, ^&&^*P<*0.01 *vs*. cortisol (62.5 nM) + AGE-BSA (200 μg/mL)-treated group. RAGE, AGEs receptor; InsR, insulin receptor; Akt, AKT serine/threonine kinase; p-Akt, phosphorylated Akt; Glut2, glucose transporter type 2; 2-NBDG, 2-Deoxy-2-[(7-nitro-2,1,3-benzoxadiazol-4-yl) amino]-D-glucose.


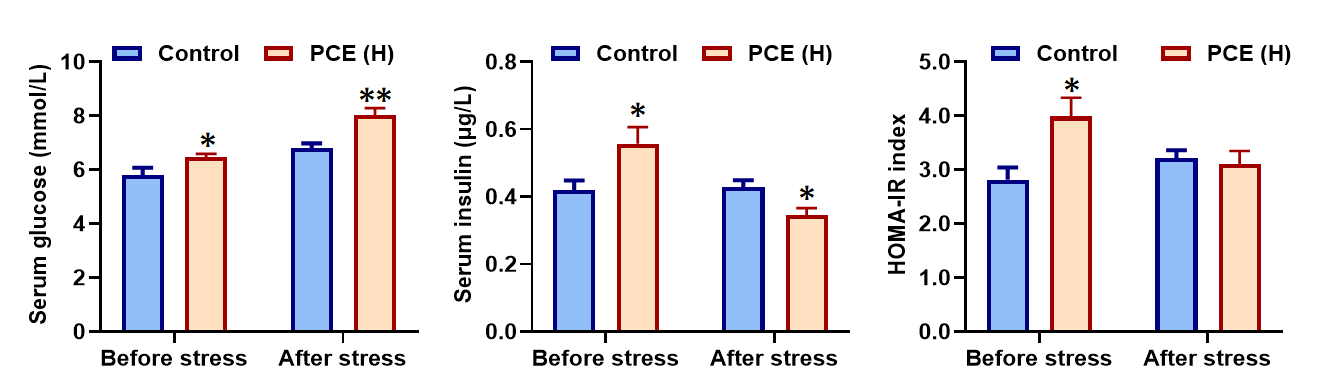


**Fig S10. Effect of PCE on serum glucose & insulin phenotype in PW52 female offspring rats before and after chronic stress.** Female offspring rats were randomly selected from each litter and allowed free access to standard feed until PW50, and was subjected to chronic stress for 14 days [5 minutes ice water swimming (5-7℃) per day from PW51 to PW52] prior to sampling. (A) Serum glucose levels; (B) Serum insulin levels; (C) HOMA-IR index. The data are shown as Mean ± S.E.M., n = 12. Two-tailed unpaired Student’s *t*-test. ^*^*P<*0.05, ^**^*P<*0.01 *vs*. control. PW, postnatal week; HOMA-IR, homeostasis model assessment of insulin resistance; PCE(H), prenatal caffeine exposure [120 mg/(kg·d)].


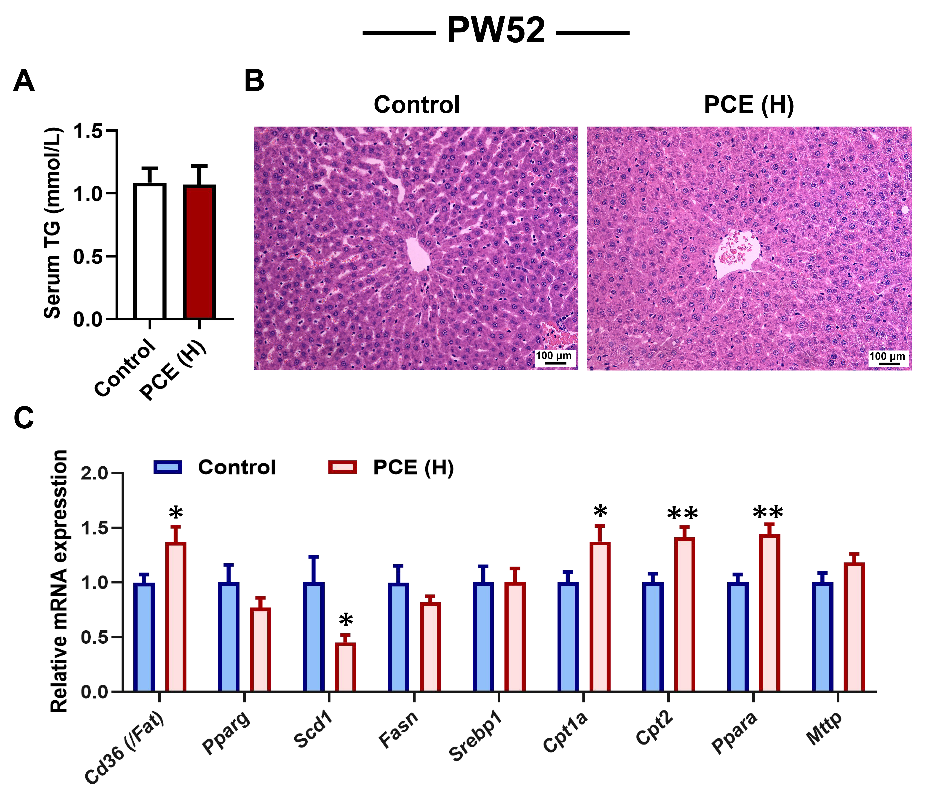


**Fig S11. Effect of PCE on serum TG levels, liver histopathology and gene expression in PW52 female offspring rats.** (A) Serum TG level; (B) Hematoxylin-eosin staining, scale bar: 100 μm; (C) mRNA expression of *Cd36*, *Pparg*, *Scd1*, *Fasn*, *Srebp1*, *Cpt1a*, *Cpt2*, *Ppara*, *Mttp*. The data are shown as Mean ± S.E.M., n = 12. Two-tailed unpaired Student’s *t*-test. ^*^*P<*0.05, ^**^*P<*0.01 *vs*. control. PW, postnatal week; TG, triglyceride; *Cd36*, fatty acid translocase, namely *Fat*; *Pparg*, peroxisome proliferator activated receptor γ; *Scd1*, stearoyl-Coenzyme A desaturase 1; *Fasn*, fatty acid synthase; *Srebp1*, sterol-regulatory element binding protein-1c; *Cpt1a*, carnitine palmitoyltransferase 1α; *Cpt2*, carnitine palmitoyltransferase 2; *Ppara*, peroxisome proliferator activated receptor α; *Mttp*, microsomal triglyceride transfer protein; PCE(H), prenatal caffeine exposure [120 mg/(kg·d)].

**Supporting Tables**

**Table S1.** Details of the main chemicals and reagents used in this study.

| **Chemicals and reagents** | **Source** |
| --- | --- |
| Caffeine (CAS No. 58-08-2) | Sigma-Aldrich (St. Louis, MO, USA) |
| Corticosterone (CAS No. 50-22-6) | Sigma-Aldrich (St. Louis, MO, USA) |
| Cortisol (CAS No. 50-23-7) | Sigma-Aldrich (St. Louis, MO, USA) |
| 2-NBDG (CAS No.186689-07-6) | Sigma-Aldrich (St. Louis, MO, USA) |
| AGE-BSA (No. 2221-BSA) and control BSA (No. 2221-10) | BioVision (Milpitas, CA, USA) |
| Rat insulin ELISA kit ((No. 10-1251-01) | Mercodia (Uppsala, Sweden) |
| Gibco™ Fetal bovine serum | Thermo Fisher Scientific Co., Ltd. (Waltham, MA, USA) |
| Gibco™ MEM α | Thermo Fisher Scientific Co., Ltd. (Waltham, MA, USA) |
| Gibco™ DMEM | Thermo Fisher Scientific Co., Ltd. (Waltham, MA, USA) |
| Gibco™ IMEM | Thermo Fisher Scientific Co., Ltd. (Waltham, MA, USA) |
| Gibco™ PBS, pH 7.4 | Thermo Fisher Scientific Co., Ltd. (Waltham, MA, USA) |
| TRIzol ^TM^ Reagent (No. 15596026) | Thermo Fisher Scientific Co., Ltd. (Waltham, MA, USA) |
| AGEs Polyclonal Antibody (No. BS-1158R) | Thermo Fisher Scientific Co., Ltd. (Waltham, MA, USA) |
| HiScript III RT SuperMix for qPCR (+gDNA wiper) (No. R323-01) | Vazyme Biotech Co., Ltd. (Nanjing, Jiangsu, China) |
| Cham Q Universal SYBR qPCR Master Mix (No. Q712-02) | Vazyme Biotech Co., Ltd. (Nanjing, Jiangsu, China) |
| miScript II RT kit (No. 218160) | Qiagen (Hilden, Germany) |
| miScript SYBR Green PCR kit (No. 218073) | Qiagen (Hilden, Germany) |
| Enhanced BCA Protein Assay Kit (No. P0009) | Beyotime Biotechnology (Shanghai, China) |
| RIPA lysis buffer (No. P0013B) | Beyotime Biotechnology (Shanghai, China) |
| PVDF membranes | Millipore Co., Ltd. (Bedford, USA) |
| ECL kit | Perkin Elmer Inc. (Boston, Mass) |
| DNA purification kit | TIANGEN Biotechnology Co., Ltd. (Beijing, China) |
| Glucose assay kit (No. F006-1-1) | Nanjing Jiancheng Bioengineering Institute (Nanjing, Jiangsu, China) |
| Rat TNF-α ELISA kit (No. E-EL-R2856) | Elabscience Biotechnology Co.,Ltd. (Wuhan, Hubei, China) |
| Rat IL-6 ELISA kit (No. E-EL-R0015) | Elabscience Biotechnology Co.,Ltd. (Wuhan, Hubei, China) |
| Corticosterone ELISA kit (No. KGE009) | R&D Systems (Minneapolis, MN, USA) |
| β-Actin Mouse mAb (No. AC004) | Abclonal (Wuhan, Hubei, China) |
| Phospho-Akt-S473 Rabbit pAb (No. AP1068) | Abclonal (Wuhan, Hubei, China) |
| Insulin Receptor Rabbit mAb (No. A19067) | Abclonal (Wuhan, Hubei, China) |
| Pan-Akt Rabbit mAb (No. A18675) | Abclonal (Wuhan, Hubei, China) |
| Mouse monoclonal Glut2 antibody (No. sc-518022) | Santa Cruz Biotechnology (California, USA) |
| Lipofectamine^®^ 3000 Reagent | Invitrogen (Carlsbad, CA, USA) |
| miR-1224 inhibits and control | Suzhou Genepharma Co., Ltd. (Suzhou, Jiangsu, China) |
| GR siRNA and control | Suzhou Genepharma Co., Ltd. (Suzhou, Jiangsu, China) |
| InsR luciferase reporter plasmid | Suzhou Genepharma Co., Ltd. (Suzhou, Jiangsu, China) |
| Dual-Luciferase^®^ Reporter (DLR™) Assay System (No. E1910) | Promega (Beijing)Biotech Co.,Ltd (Beijing, China) |
| Rat AGEs ELISA Kit (No. CSB-E09413r) | Cusabio Biotech Co., Ltd (Wuhan, Hubei, China) |
| Omni-Easy One-Step PAGE Gel Fast Preparation Kit (No. PG212) | Shanghai Epizyme Biomedical Technology Co., Ltd. (Shanghai, China) |

ELISA, enzyme-linked immunosorbent assay; 2-NBDG, 2-Deoxy-2-[(7-nitro-2,1,3-benzoxadiazol-4-yl) amino]-D-glucose; BCA, Bicinchoninic acid; MEM α, Minimum Essential Medium α; IMEM, Improved MEM; DMEM, Dulbecco's Modified Eagle Medium; PBS, phosphate buffered saline; RIPA, Radio-immunoprecipitation assay; PVDF, polyvinylidene fluoride; ECL, electrochemiluminescence; DNA, deoxyriboNucleic acid; IL-6, interleukin 6; TNF-α, tumor necrosis factor α; AGEs, advanced glycation end products; GR, glucocorticoid receptor.

**Table S2. Primers used for RT-qPCR.**

| **Genes** | **Forward primer (5’→3’)** | **Reverse primer (5’→3’)** |
| --- | --- | --- |
| **Human:** |  |  |
| *β-actin* | CATGTACGTTGCTATCCAGGC | CTCCTTAATGTCACGCACGAT |
| *Akt1* | CAAGTCCTTGCTTTCAGGGC | ATACCTGGTGTCAGTCTCCGA |
| *Akt2* | CAGACGAGAGGGAGGAGTGGATG | CTGGGGGAGCCACACTTGTAGTC |
| *Glut2 (/Slc2a2)* | TGGGACCCTGGTTTTCACTG | TTATTACCTGTTGAGGTGCATTG |
| *GR(/NR3C1)* | CCCGTTGGTTCCGAAAATTG | AGCTTACATCTGGTCTCATGC |
| *InsR* | AAAACGAGGCCCGAAGATTTC | GAGCCCATAGACCCGGAAG |
| *IL-6* | ATGAACTCCTTCTCCACAAGC | AAGAGCCCTCAGGCTGGACTG |
| *RAGE* | CCTGGAAGGAAGCAGGATGG | GGAGACAGGACCTTCCAAGC |
| *TNF-α* | CTGGTATGAGCCCATCTATCT | GGGCAATGATCCCAAAGTAG |
| **Rat:** |  |  |
| *β-actin* | GTTGCCAATAGTGATGACCT | GGACCTGACAGACTACCTCA |
| *Akt1* | TCTATGGCGCTGAGATTGTG | CTTAATGTGCCCGTCCTTGT |
| *Akt2* | CCTCATCCCATATCCAGTTTC | TAACCTCACTCTCCATCCTC |
| *Glut2 (/Slc2a2)* | CACCCAGGAGGATCATTTATTC | AGCAGCCTCTGGTTTACT |
| *GR(/Nr3c1)* | CACCCATGACCCTGTCAGTC | AAAGCCTCCCTCTGCTAACC |
| *InsR* | TTCATTCAGGAAGACCTTCGA | AGGCCAGAGCTGACAAGTGAC |
| *Igf1* | GACCAAGGGGCTTTTACTTCAAC | TTTGTAGGCTTCAGCGGAGCAC |
| *RAGE* | CAACTACCGAGTCCGAGTCTACCA | AGAGGTTTCCCATCCAAGTGC |
| *Srebp1* | CGCCCATCGGTTTAAGGACT | ACACTCGTTTCTTTCGGGCT |
| *Fasn* | TGGCTCAGCATGGCCGCTTC | CAGCTGTCGTTGGCCCCCTC |
| *Cd36* | CCAGAACCCAGACAACCACT | CACAGGCTTTCCTTCTTTGC |
| *Scd1* | GTCAGCACCTTCTTGAGATAC | AGGATGTTCTCCCGAGATT |
| *Mttp* | AGATGTGCAACGTGGTATTC | CCTGACTAGGCTCGACTTTA |
| *Ppara* | GCTGAAGTACGGTGTGTATG | TAGGAACTCTCGGGTGATG |
| *Pparg* | TGTGGACCTCTCTGTGATGG | CATTGGGTCAGCTCTTGTGA |
| *Cpt1a* | CCTTTCCTGAAGGAGGTATTG | AGGACACATAGTCAGGGTT |
| *Cpt12* | CAACTCGTATACCCAGACCCAATC | GTTCCCATCTTGATCGAGGACATC |
| *Pck1* | CCCGAAGGCAAGAAGAAATA | GTCATCACCCACACATTCA |
| *G6pc* | GCAGGTGTATACTACGTTATGG | CAGTATCCCAACCACAAGAC |

*Akt1*, AKT serine/threonine kinase 1; *Akt2*, AKT serine/threonine kinase 2; *Glut2 (/Slc2a2)*, glucose transporter 2; GR *(/Nr3c1)*, glucocorticoid receptor; *InsR*, insulin receptor; *Igf1*, insulin growth factor 1; *IL-6*, interleukin-6; *RAGE*, AGEs receptor; *TNF-α*, tumor necrosis factor α.

**Table S3. Hepatic miRNAs expression in PCE female fetal rats.**

| **No.** | **ID** | | **CPM** | | **Log_2_(FC)** | **No.** | **ID** | **CPM** | | **Log_2_(FC)** |
| --- | --- | --- | --- | --- | --- | --- | --- | --- | --- | --- |
|  |  |  | **Control** | **PPE(H)** |  |  |  | **Control** | **PPE(H)** |  |
| 1 | rno-miR-291a-3p | 0.8 | | 37.1 | 5.54 | 61 | rno-miR-672-5p | 6.6 | 15.3 | 1.21 |
| 2 | 15_36839 | 0.8 | | 21.8 | 4.77 | 62 | rno-miR-296-3p | 60.4 | 139 | 1.20 |
| 3 | 3_10505 | 0.8 | | 15.3 | 4.26 | 63 | rno-miR-350 | 18.2 | 41.5 | 1.19 |
| 4 | rno-miR-324-3p | 0.8 | | 13.8 | 4.11 | 64 | rno-miR-433-5p | 17.4 | 39.3 | 1.18 |
| 5 | rno-miR-877 | 0.8 | | 13.1 | 4.03 | 65 | rno-miR-1843b-5p | 7.4 | 16.7 | 1.17 |
| 6 | rno-miR-1298 | 5 | | 74.2 | 3.89 | 66 | rno-miR-210-5p | 7.4 | 16.7 | 1.17 |
| 7 | rno-miR-149-5p | 2.5 | | 37.1 | 3.89 | 67 | 2_5581 | 13.2 | 29.1 | 1.14 |
| 8 | rno-miR-295-3p | 1.7 | | 22.6 | 3.73 | 68 | rno-miR-335 | 123.2 | 270.7 | 1.14 |
| 9 | rno-let-7c-2-3p | 0.8 | | 7.3 | 3.19 | 69 | rno-miR-503-3p | 52.9 | 115 | 1.12 |
| 10 | rno-miR-490-5p | 0.8 | | 7.3 | 3.19 | 70 | 2_7300 | 9.1 | 19.6 | 1.11 |
| 11 | rno-miR-484 | 4.1 | | 31.3 | 2.93 | 71 | rno-miR-30c-2-3p | 269.5 | 573.4 | 1.09 |
| 12 | rno-miR-142-3p | 11.6 | | 83 | 2.84 | 72 | rno-miR-196a-5p | 13.2 | 27.7 | 1.07 |
| 13 | rno-miR-193a-5p | 3.3 | | 20.4 | 2.63 | 73 | rno-miR-501-3p | 87.6 | 179.7 | 1.04 |
| 14 | rno-miR-1b | 324.9 | | 1873.1 | 2.53 | 74 | rno-miR-758-3p | 100.9 | 205.2 | 1.02 |
| 15 | 12_32681_star | 0.8 | | 4.4 | 2.46 | 75 | 3_10819 | 14.1 | 28.4 | 1.01 |
| 16 | rno-miR-871-3p | 0.8 | | 4.4 | 2.46 | 76 | rno-miR-30b-3p | 325.7 | 654.9 | 1.01 |
| 17 | rno-miR-665 | 11.6 | | 61.9 | 2.42 | 77 | X_44874 | 73.6 | 36.4 | -1.02 |
| 18 | 2_6500 | 1.7 | | 8.7 | 2.36 | 78 | rno-miR-99b-3p | 135.6 | 66.9 | -1.02 |
| 19 | rno-miR-708-3p | 2.5 | | 12.4 | 2.31 | 79 | rno-miR-425-5p | 115.7 | 56 | -1.05 |
| 20 | rno-miR-22-5p | 12.4 | | 59.7 | 2.27 | 80 | rno-miR-485-5p | 151.3 | 70.6 | -1.10 |
| 21 | 6_19658 | 4.1 | | 18.9 | 2.20 | 81 | rno-miR-29b-3p | 3.3 | 1.5 | -1.14 |
| 22 | rno-miR-598-3p | 5.8 | | 26.2 | 2.18 | 82 | rno-miR-203a-3p | 23.1 | 10.2 | -1.18 |
| 23 | rno-miR-9a-3p | 0.8 | | 3.6 | 2.17 | 83 | rno-miR-363-3p | 13.2 | 5.8 | -1.19 |
| 24 | rno-miR-1-3p | 311.7 | | 1399.4 | 2.17 | 84 | rno-miR-151-5p | 134.8 | 58.9 | -1.19 |
| 25 | rno-miR-409a-3p | 15.7 | | 66.9 | 2.09 | 85 | 17_40739 | 106.6 | 45.1 | -1.24 |
| 26 | 7_22250 | 16.5 | | 69.9 | 2.08 | 86 | 4_14148 | 39.7 | 16.7 | -1.25 |
| 27 | 17_40212_star | 13.2 | | 53.8 | 2.03 | 87 | rno-miR-30e-5p | 379.5 | 156.5 | -1.28 |
| 28 | rno-miR-144-5p | 24.8 | | 96.8 | 1.96 | 88 | 11_30620 | 1.7 | 0.7 | -1.28 |
| 29 | 7_22921 | 31.4 | | 121.5 | 1.95 | 89 | 4_12920 | 38.9 | 16 | -1.28 |
| 30 | rno-miR-345-3p | 46.3 | | 178.3 | 1.95 | 90 | 17_40738 | 65.3 | 26.2 | -1.32 |
| 31 | rno-miR-292-3p | 0.8 | | 2.9 | 1.86 | 91 | 2_6800 | 25.6 | 10.2 | -1.33 |
| 32 | 19_43528 | 2.5 | | 8.7 | 1.80 | 92 | rno-miR-134-3p | 20.7 | 8 | -1.37 |
| 33 | rno-miR-218a-5p | 10.7 | | 37.1 | 1.79 | 93 | rno-miR-200b-5p | 27.3 | 10.2 | -1.42 |
| 34 | 17_40817 | 14.1 | | 48.8 | 1.79 | 94 | 13_34558 | 19.8 | 7.3 | -1.44 |
| 35 | rno-miR-221-3p | 151.3 | | 506.5 | 1.74 | 95 | rno-miR-92b-3p | 26.5 | 9.5 | -1.48 |
| 36 | 15_37552 | 29.8 | | 97.5 | 1.71 | 96 | rno-miR-543-3p | 238.9 | 83 | -1.53 |
| 37 | rno-miR-206-3p | 2271 | | 7427.6 | 1.71 | 97 | rno-miR-125a-3p | 57 | 19.6 | -1.54 |
| 38 | rno-miR-130b-5p | 14.1 | | 45.8 | 1.70 | 98 | rno-miR-32-5p | 21.5 | 7.3 | -1.56 |
| 39 | rno-miR-674-5p | 53.7 | | 170.3 | 1.67 | 99 | rno-miR-19b-3p | 37.2 | 11.6 | -1.68 |
| 40 | rno-miR-339-5p | 29.8 | | 92.4 | 1.63 | 100 | rno-miR-370-5p | 60.4 | 18.2 | -1.73 |
| 41 | rno-miR-466c-5p | 14.1 | | 43.7 | 1.63 | 101 | rno-miR-215 | 43.8 | 13.1 | -1.74 |
| **42** | **rno-miR-1224** | **21.5** | | **66.2** | **1.62** | 102 | rno-miR-184 | 489.4 | 140.4 | -1.80 |
| 43 | rno-miR-540-3p | 318.3 | | 969.3 | 1.61 | 103 | rno-miR-181a-1-3p | 73.6 | 21.1 | -1.80 |
| 44 | rno-miR-130a-3p | 36.4 | | 109.9 | 1.59 | 104 | rno-miR-223-3p | 2.5 | 0.7 | -1.84 |
| 45 | rno-miR-154-5p | 110 | | 320.9 | 1.54 | 105 | rno-miR-221-5p | 37.2 | 10.2 | -1.87 |
| 46 | rno-miR-298-5p | 78.5 | | 219 | 1.48 | 106 | rno-miR-34a-5p | 56.2 | 14.6 | -1.94 |
| 47 | 14_36156 | 0.8 | | 2.2 | 1.46 | 107 | rno-miR-3544 | 17.4 | 4.4 | -1.98 |
| 48 | rno-miR-224-5p | 0.8 | | 2.2 | 1.46 | 108 | rno-miR-140-5p | 33.9 | 8 | -2.08 |
| 49 | rno-miR-532-3p | 28.9 | | 75.7 | 1.39 | 109 | 12_32062 | 157.9 | 32.7 | -2.27 |
| 50 | rno-miR-3589 | 2.5 | | 6.5 | 1.38 | 110 | 10_27486 | 19.8 | 3.6 | -2.46 |
| 51 | rno-miR-133a-3p | 8.3 | | 21.1 | 1.35 | 111 | 1_1705 | 453 | 73.5 | -2.62 |
| 52 | rno-miR-3547 | 6.6 | | 16.7 | 1.34 | 112 | rno-miR-466c-3p | 74.4 | 11.6 | -2.68 |
| 53 | 14_35179 | 10.7 | | 26.9 | 1.33 | 113 | rno-miR-222-3p | 44.6 | 6.5 | -2.78 |
| 54 | rno-miR-106b-5p | 19.8 | | 49.5 | 1.32 | 114 | rno-miR-1843a-5p | 57 | 8 | -2.83 |
| 55 | rno-miR-872-5p | 38.9 | | 96.1 | 1.30 | 115 | rno-miR-3068-5p | 39.7 | 4.4 | -3.17 |
| 56 | rno-miR-667-5p | 14.9 | | 35.7 | 1.26 | 116 | rno-miR-27b-5p | 33.9 | 3.6 | -3.24 |
| 57 | rno-miR-18a-5p | 20.7 | | 49.5 | 1.26 | 117 | rno-miR-1249 | 36.4 | 2.2 | -4.05 |
| 58 | 17_40741_star | 14.1 | | 33.5 | 1.25 | 118 | rno-miR-195-3p | 14.9 | 0.7 | -4.41 |
| 59 | 17_40787 | 20.7 | | 48.8 | 1.24 | 119 | 17_39199 | 15.7 | 0.7 | -4.49 |
| 60 | rno-miR-3473 | 48.8 | | 115 | 1.24 | 120 | 5_15886 | 20.7 | 0.7 | -4.89 |

FC, fold change; CPM, counts per million; PCE(H), prenatal caffeine exposure [120 mg/(kg·d)].
